# Supplementary material for: Analyzing pre-symptomatic tissue to gain insights into the molecular and mechanistic origins of late-onset degenerative trinucleotide repeat disease
Source: Nucleic Acids Res. 2020 May 28;48(12):6740–58. doi: 10.1093/nar/gkaa422 (PMC7337964; doi:10.1093/nar/gkaa422)
Supplement: gkaa422_Supplemental_File [file gkaa422_supplemental_file.pptx]

## Slide 1
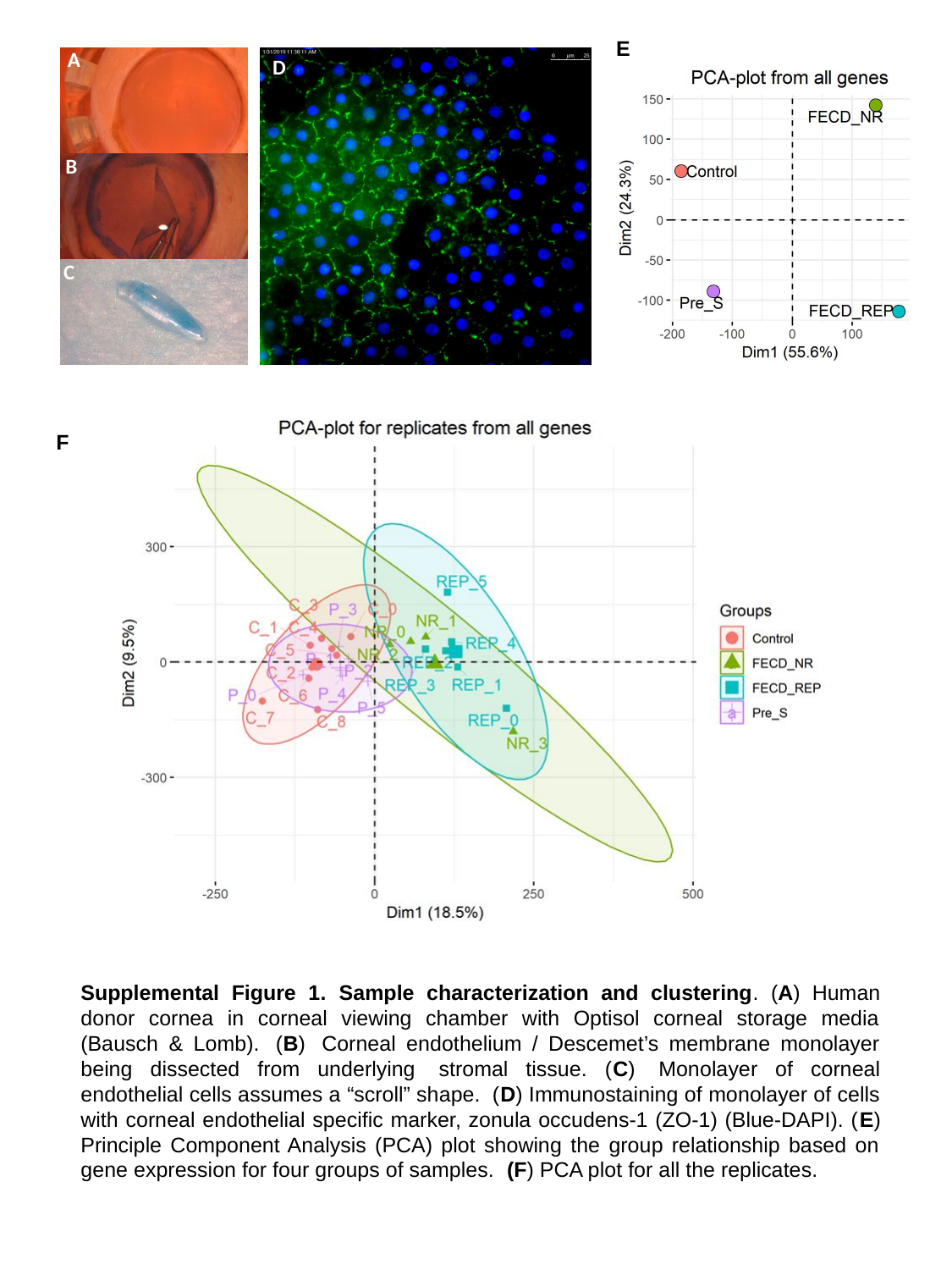

E
A
D
B
C
F
Supplemental Figure 1. Sample characterization and clustering. (A) Human donor cornea in corneal viewing chamber with Optisol corneal storage media (Bausch & Lomb).  (B)  Corneal endothelium / Descemet’s membrane monolayer being dissected from underlying  stromal tissue. (C)  Monolayer of corneal endothelial cells assumes a “scroll” shape.  (D) Immunostaining of monolayer of cells with corneal endothelial specific marker, zonula occudens-1 (ZO-1) (Blue-DAPI). (E) Principle Component Analysis (PCA) plot showing the group relationship based on gene expression for four groups of samples. (F) PCA plot for all the replicates.

## Slide 2
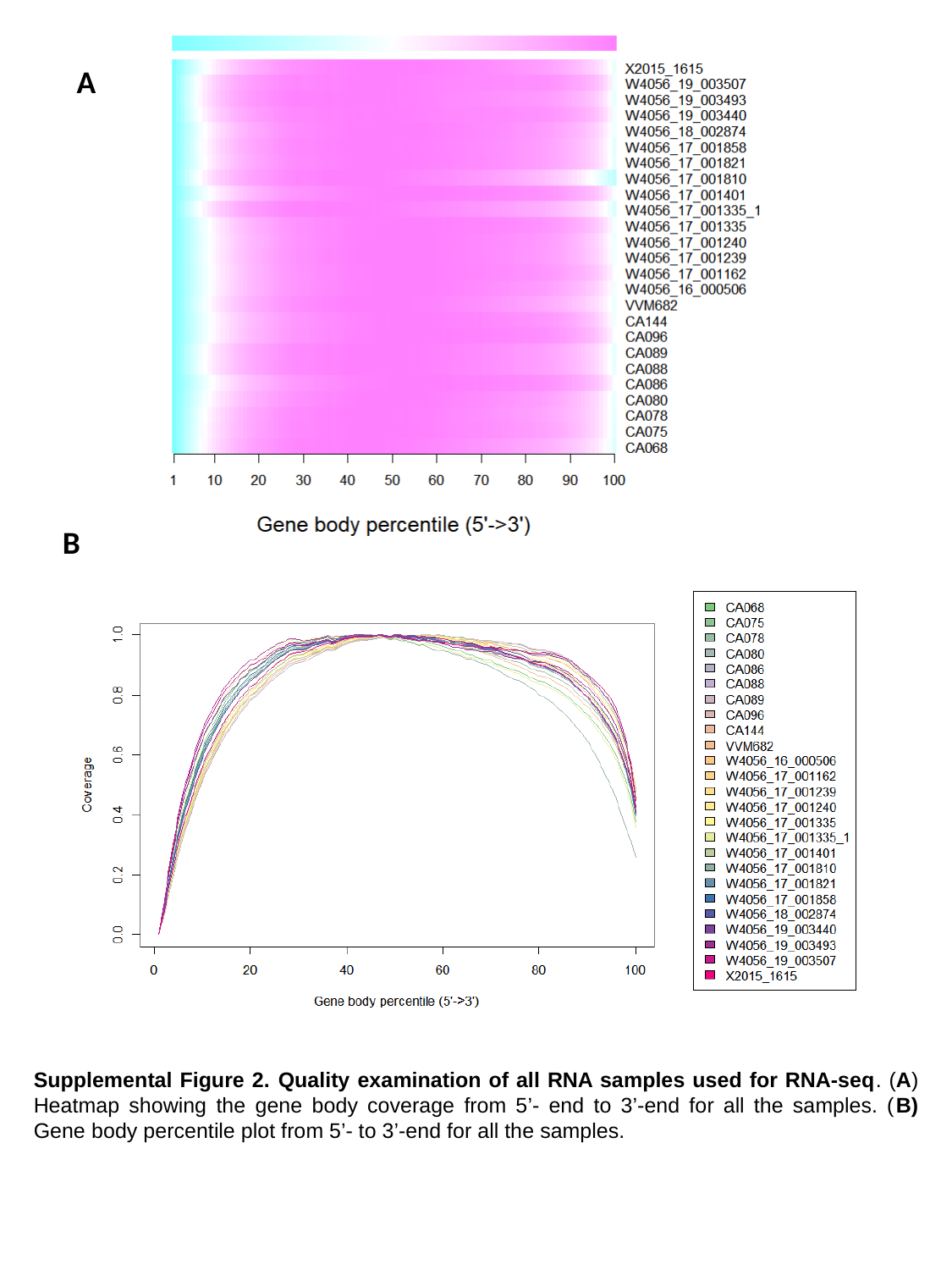

A
B
Supplemental Figure 2. Quality examination of all RNA samples used for RNA-seq. (A) Heatmap showing the gene body coverage from 5’- end to 3’-end for all the samples. (B) Gene body percentile plot from 5’- to 3’-end for all the samples.

## Slide 3
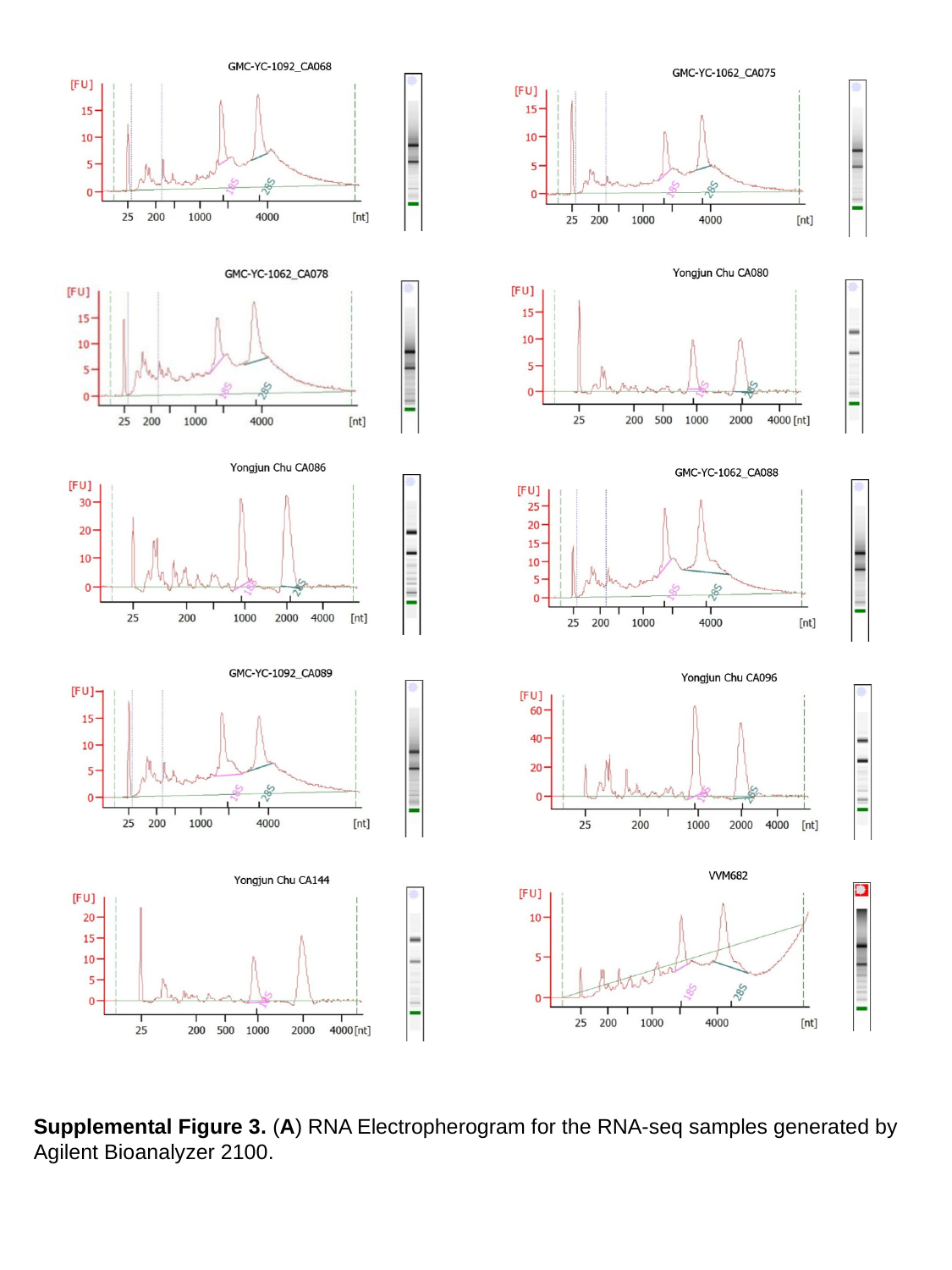

Supplemental Figure 3. (A) RNA Electropherogram for the RNA-seq samples generated by Agilent Bioanalyzer 2100.

## Slide 4
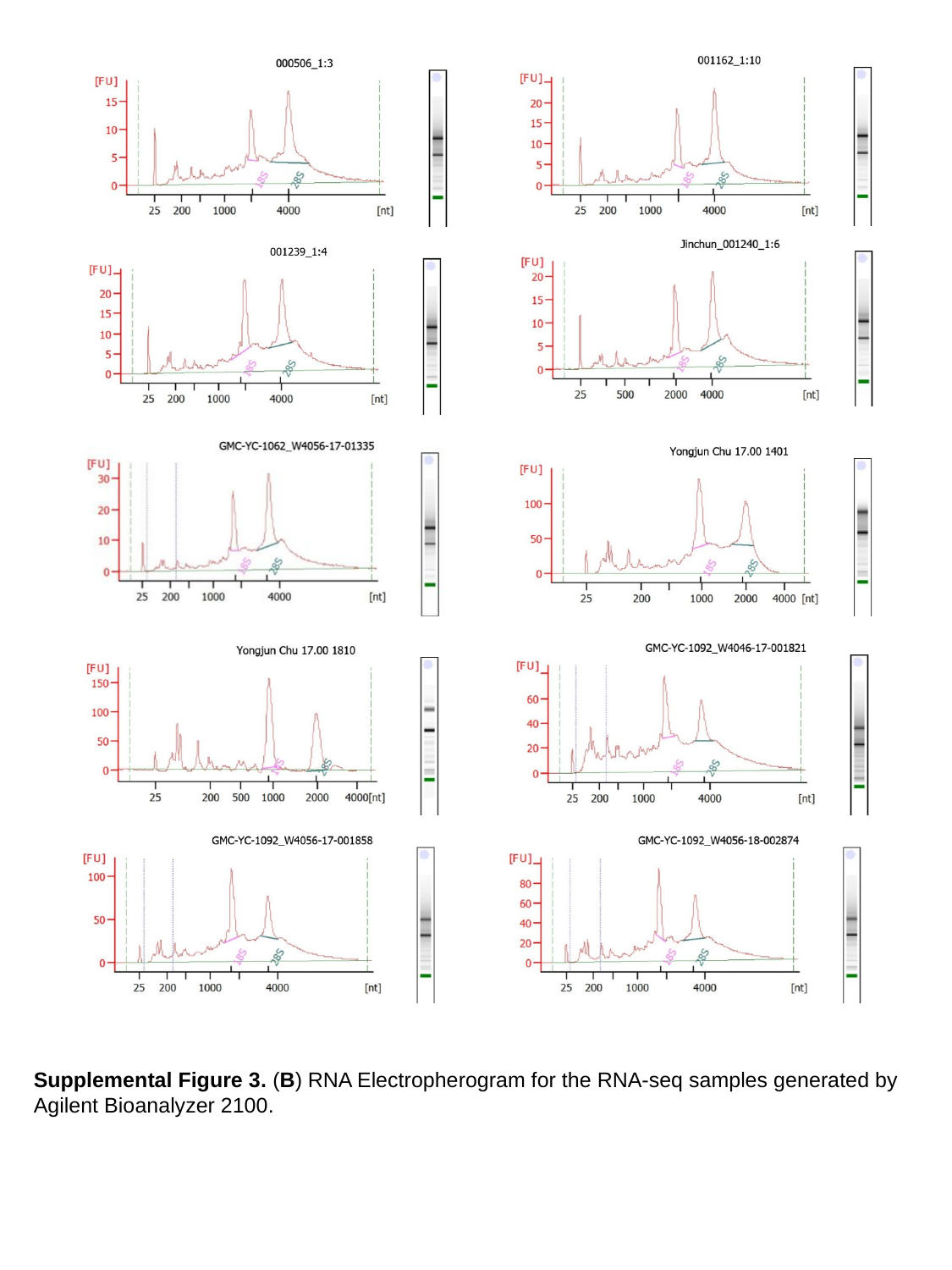

Supplemental Figure 3. (B) RNA Electropherogram for the RNA-seq samples generated by Agilent Bioanalyzer 2100.

## Slide 5
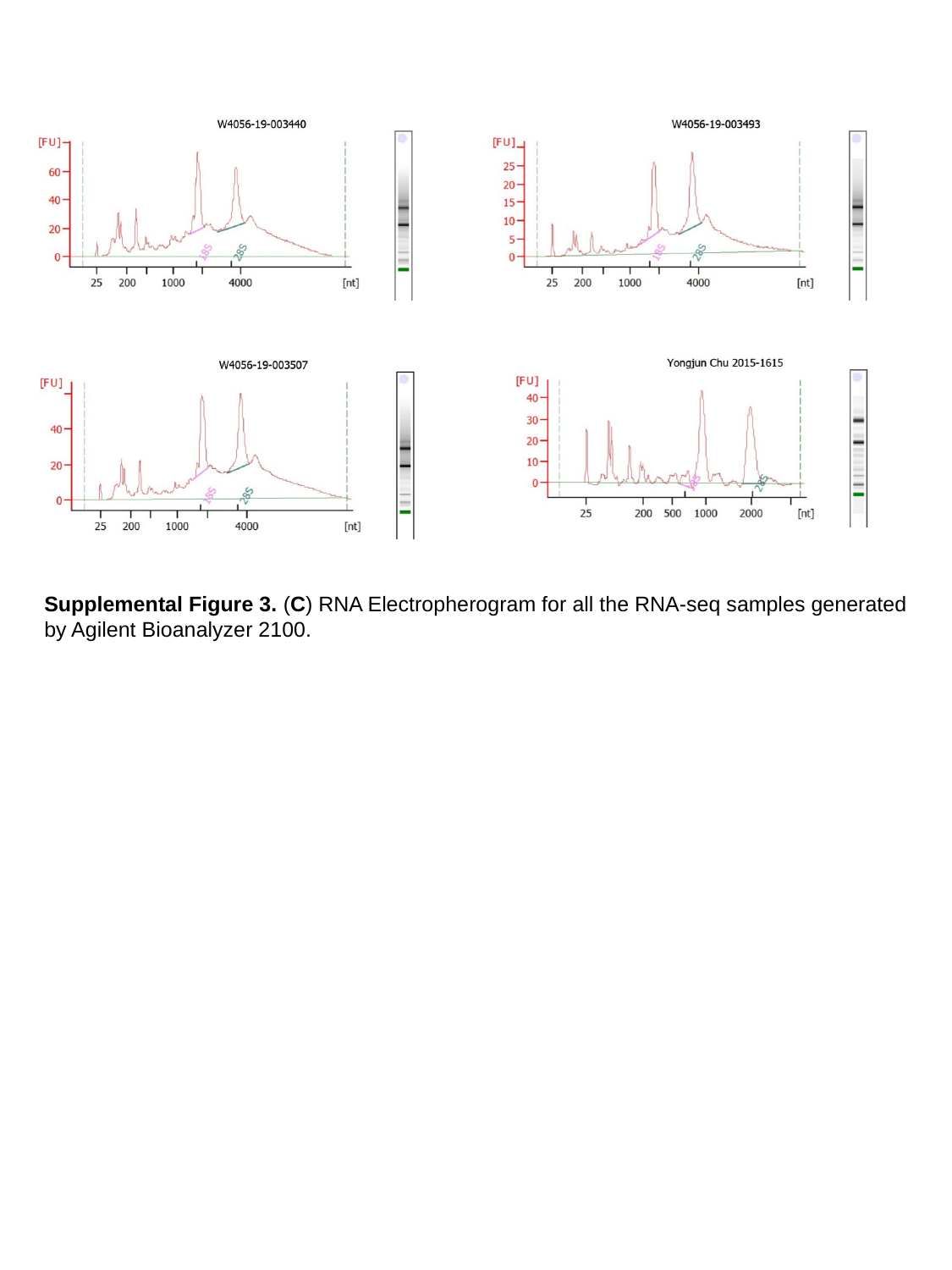

Supplemental Figure 3. (C) RNA Electropherogram for all the RNA-seq samples generated by Agilent Bioanalyzer 2100.

## Slide 6
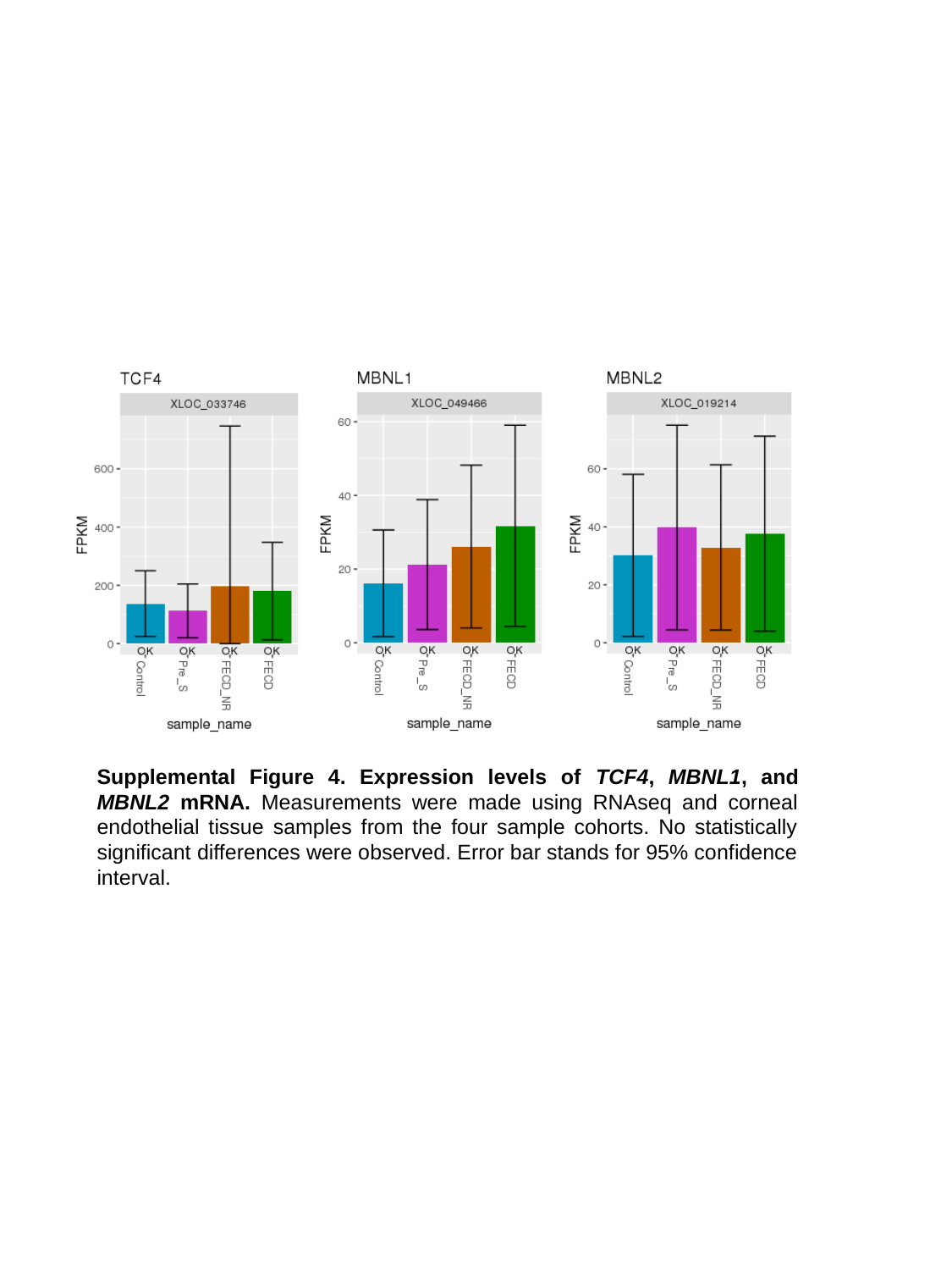

Supplemental Figure 4. Expression levels of TCF4, MBNL1, and MBNL2 mRNA. Measurements were made using RNAseq and corneal endothelial tissue samples from the four sample cohorts. No statistically significant differences were observed. Error bar stands for 95% confidence interval.

## Slide 7
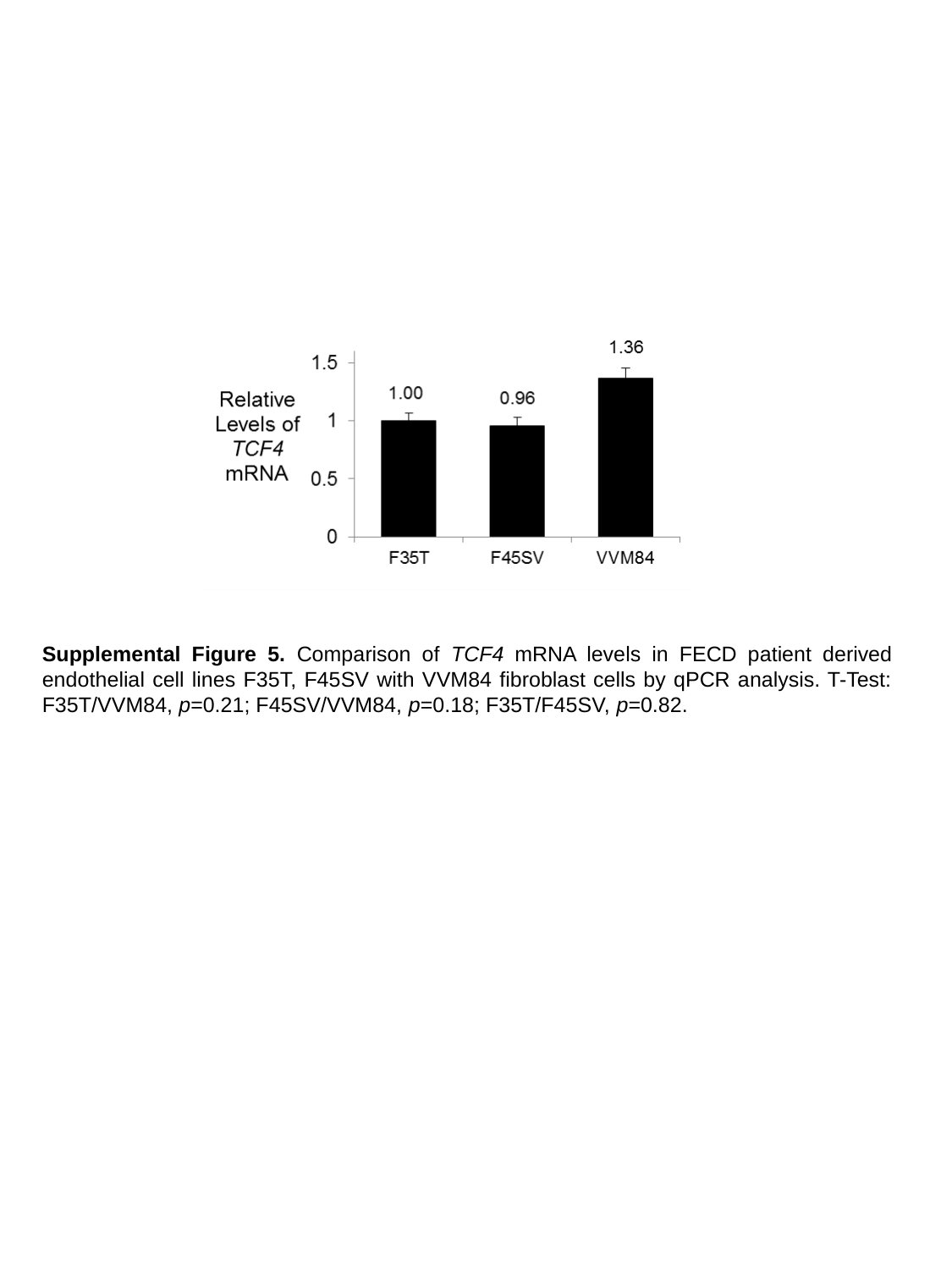

Supplemental Figure 5. Comparison of TCF4 mRNA levels in FECD patient derived endothelial cell lines F35T, F45SV with VVM84 fibroblast cells by qPCR analysis. T-Test: F35T/VVM84, p=0.21; F45SV/VVM84, p=0.18; F35T/F45SV, p=0.82.

## Slide 8
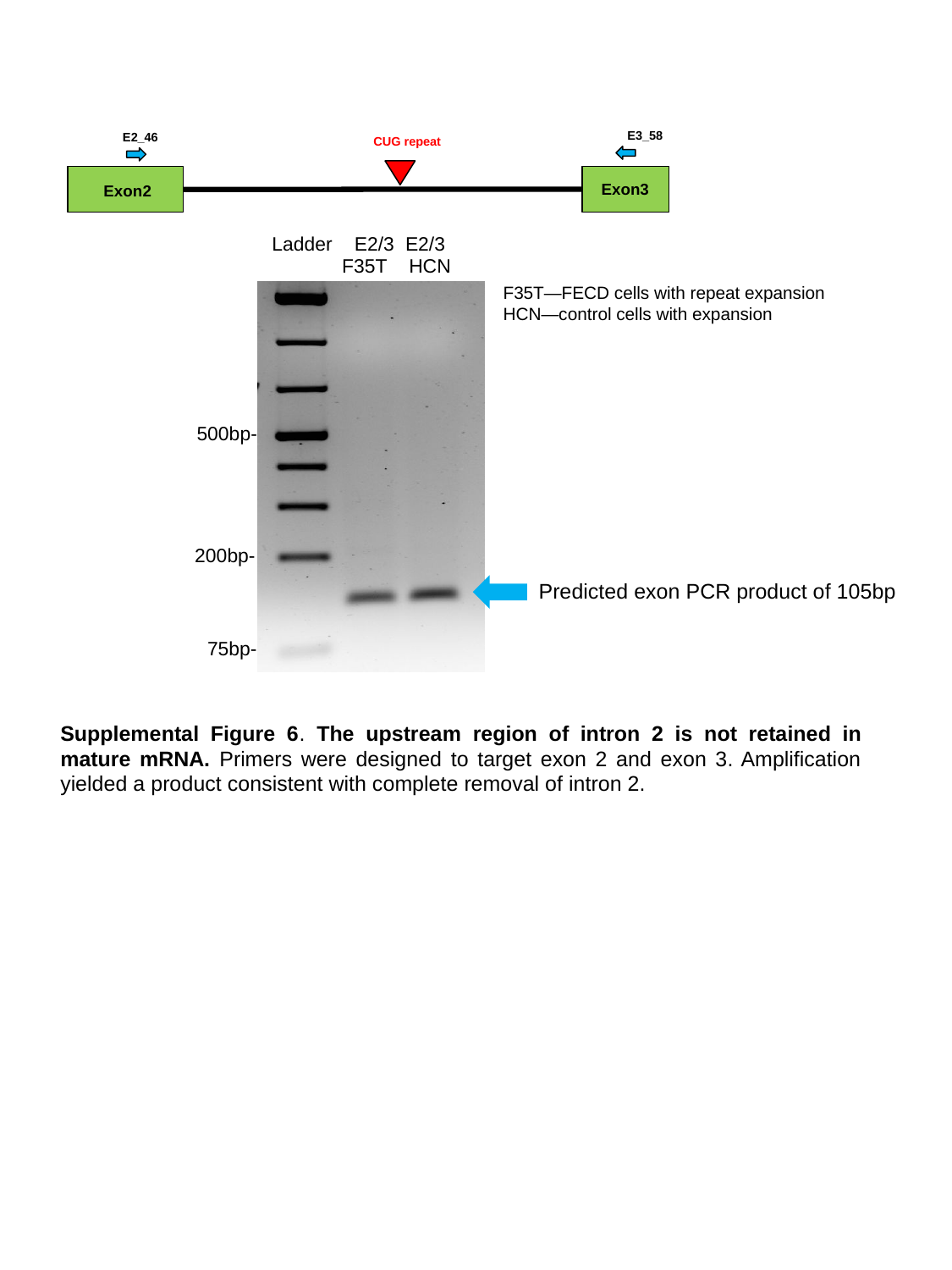

E3_58
E2_46
CUG repeat
Exon3
Exon2
Ladder E2/3 E2/3
F35T HCN
F35T—FECD cells with repeat expansion
HCN—control cells with expansion
500bp-
200bp-
Predicted exon PCR product of 105bp
75bp-
Supplemental Figure 6. The upstream region of intron 2 is not retained in mature mRNA. Primers were designed to target exon 2 and exon 3. Amplification yielded a product consistent with complete removal of intron 2.

## Slide 9
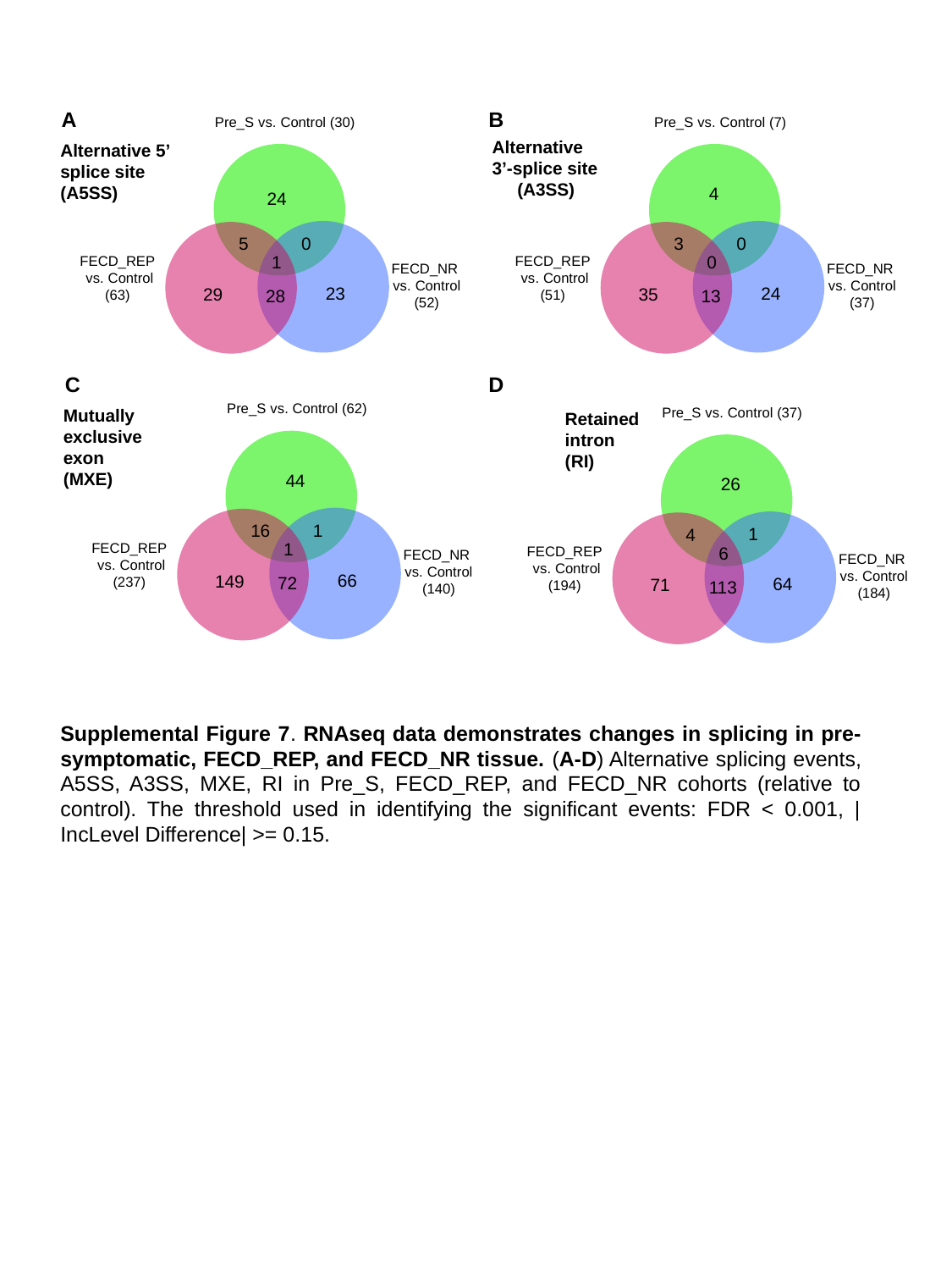

A
B
Pre_S vs. Control (30)
Pre_S vs. Control (7)
Alternative 3’-splice site
(A3SS)
Alternative 5’ splice site
(A5SS)
4
24
0
0
5
3
1
0
FECD_REP
 vs. Control
(63)
FECD_REP
 vs. Control
(51)
FECD_NR
vs. Control
(52)
FECD_NR
vs. Control
(37)
23
24
29
35
28
13
C
D
Pre_S vs. Control (62)
Pre_S vs. Control (37)
Mutually exclusive exon
(MXE)
Retained
intron
(RI)
44
26
1
16
1
4
1
FECD_REP
 vs. Control
(237)
6
FECD_REP
 vs. Control
(194)
FECD_NR
vs. Control
(140)
FECD_NR
vs. Control
(184)
66
149
72
64
71
113
Supplemental Figure 7. RNAseq data demonstrates changes in splicing in pre-symptomatic, FECD_REP, and FECD_NR tissue. (A-D) Alternative splicing events, A5SS, A3SS, MXE, RI in Pre_S, FECD_REP, and FECD_NR cohorts (relative to control). The threshold used in identifying the significant events: FDR < 0.001, |IncLevel Difference| >= 0.15.

## Slide 10
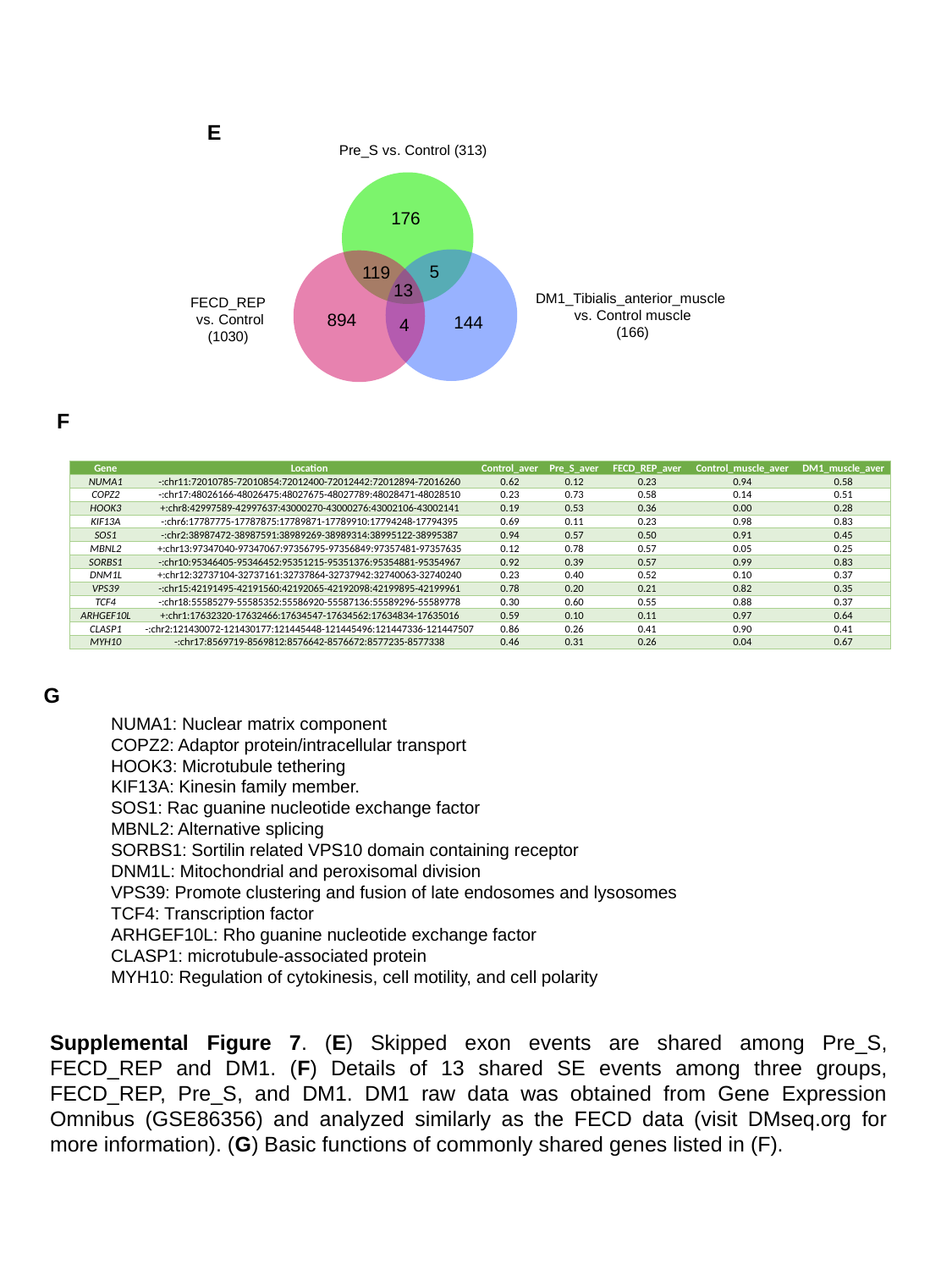

E
Pre_S vs. Control (313)
176
5
119
13
DM1_Tibialis_anterior_muscle
vs. Control muscle
(166)
FECD_REP
 vs. Control
(1030)
894
144
4
F
| Gene | Location | Control\_aver | Pre\_S\_aver | FECD\_REP\_aver | Control\_muscle\_aver | DM1\_muscle\_aver |
| --- | --- | --- | --- | --- | --- | --- |
| NUMA1 | -:chr11:72010785-72010854:72012400-72012442:72012894-72016260 | 0.62 | 0.12 | 0.23 | 0.94 | 0.58 |
| COPZ2 | -:chr17:48026166-48026475:48027675-48027789:48028471-48028510 | 0.23 | 0.73 | 0.58 | 0.14 | 0.51 |
| HOOK3 | +:chr8:42997589-42997637:43000270-43000276:43002106-43002141 | 0.19 | 0.53 | 0.36 | 0.00 | 0.28 |
| KIF13A | -:chr6:17787775-17787875:17789871-17789910:17794248-17794395 | 0.69 | 0.11 | 0.23 | 0.98 | 0.83 |
| SOS1 | -:chr2:38987472-38987591:38989269-38989314:38995122-38995387 | 0.94 | 0.57 | 0.50 | 0.91 | 0.45 |
| MBNL2 | +:chr13:97347040-97347067:97356795-97356849:97357481-97357635 | 0.12 | 0.78 | 0.57 | 0.05 | 0.25 |
| SORBS1 | -:chr10:95346405-95346452:95351215-95351376:95354881-95354967 | 0.92 | 0.39 | 0.57 | 0.99 | 0.83 |
| DNM1L | +:chr12:32737104-32737161:32737864-32737942:32740063-32740240 | 0.23 | 0.40 | 0.52 | 0.10 | 0.37 |
| VPS39 | -:chr15:42191495-42191560:42192065-42192098:42199895-42199961 | 0.78 | 0.20 | 0.21 | 0.82 | 0.35 |
| TCF4 | -:chr18:55585279-55585352:55586920-55587136:55589296-55589778 | 0.30 | 0.60 | 0.55 | 0.88 | 0.37 |
| ARHGEF10L | +:chr1:17632320-17632466:17634547-17634562:17634834-17635016 | 0.59 | 0.10 | 0.11 | 0.97 | 0.64 |
| CLASP1 | -:chr2:121430072-121430177:121445448-121445496:121447336-121447507 | 0.86 | 0.26 | 0.41 | 0.90 | 0.41 |
| MYH10 | -:chr17:8569719-8569812:8576642-8576672:8577235-8577338 | 0.46 | 0.31 | 0.26 | 0.04 | 0.67 |
G
NUMA1: Nuclear matrix component
COPZ2: Adaptor protein/intracellular transport
HOOK3: Microtubule tethering
KIF13A: Kinesin family member.
SOS1: Rac guanine nucleotide exchange factor
MBNL2: Alternative splicing
SORBS1: Sortilin related VPS10 domain containing receptor
DNM1L: Mitochondrial and peroxisomal division
VPS39: Promote clustering and fusion of late endosomes and lysosomes
TCF4: Transcription factor
ARHGEF10L: Rho guanine nucleotide exchange factor
CLASP1: microtubule-associated protein
MYH10: Regulation of cytokinesis, cell motility, and cell polarity
Supplemental Figure 7. (E) Skipped exon events are shared among Pre_S, FECD_REP and DM1. (F) Details of 13 shared SE events among three groups, FECD_REP, Pre_S, and DM1. DM1 raw data was obtained from Gene Expression Omnibus (GSE86356) and analyzed similarly as the FECD data (visit DMseq.org for more information). (G) Basic functions of commonly shared genes listed in (F).

## Slide 11
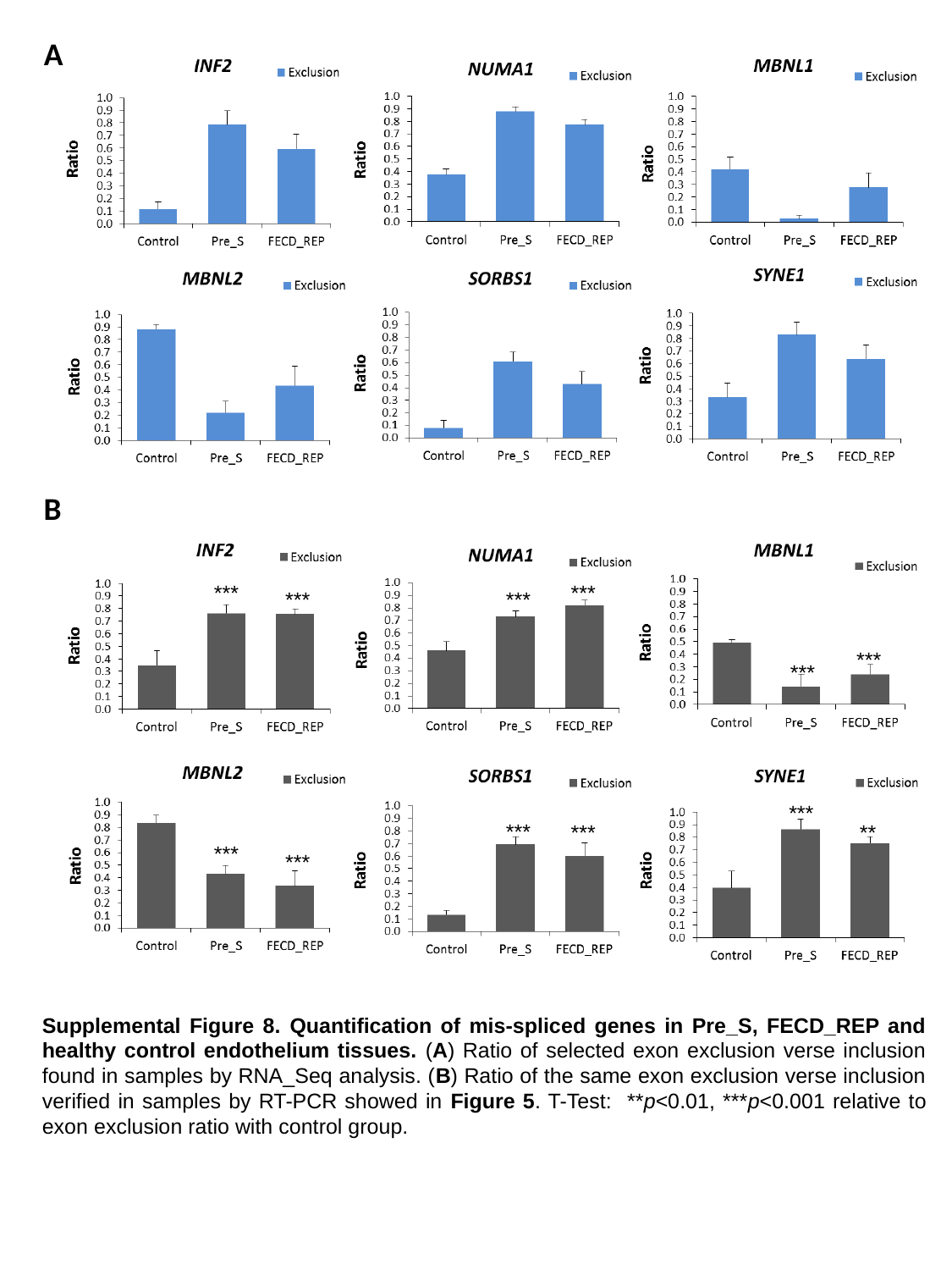

A
B
***
***
***
***
***
***
***
***
***
**
***
***
Supplemental Figure 8. Quantification of mis-spliced genes in Pre_S, FECD_REP and healthy control endothelium tissues. (A) Ratio of selected exon exclusion verse inclusion found in samples by RNA_Seq analysis. (B) Ratio of the same exon exclusion verse inclusion verified in samples by RT-PCR showed in Figure 5. T-Test: **p<0.01, ***p<0.001 relative to exon exclusion ratio with control group.

## Slide 12
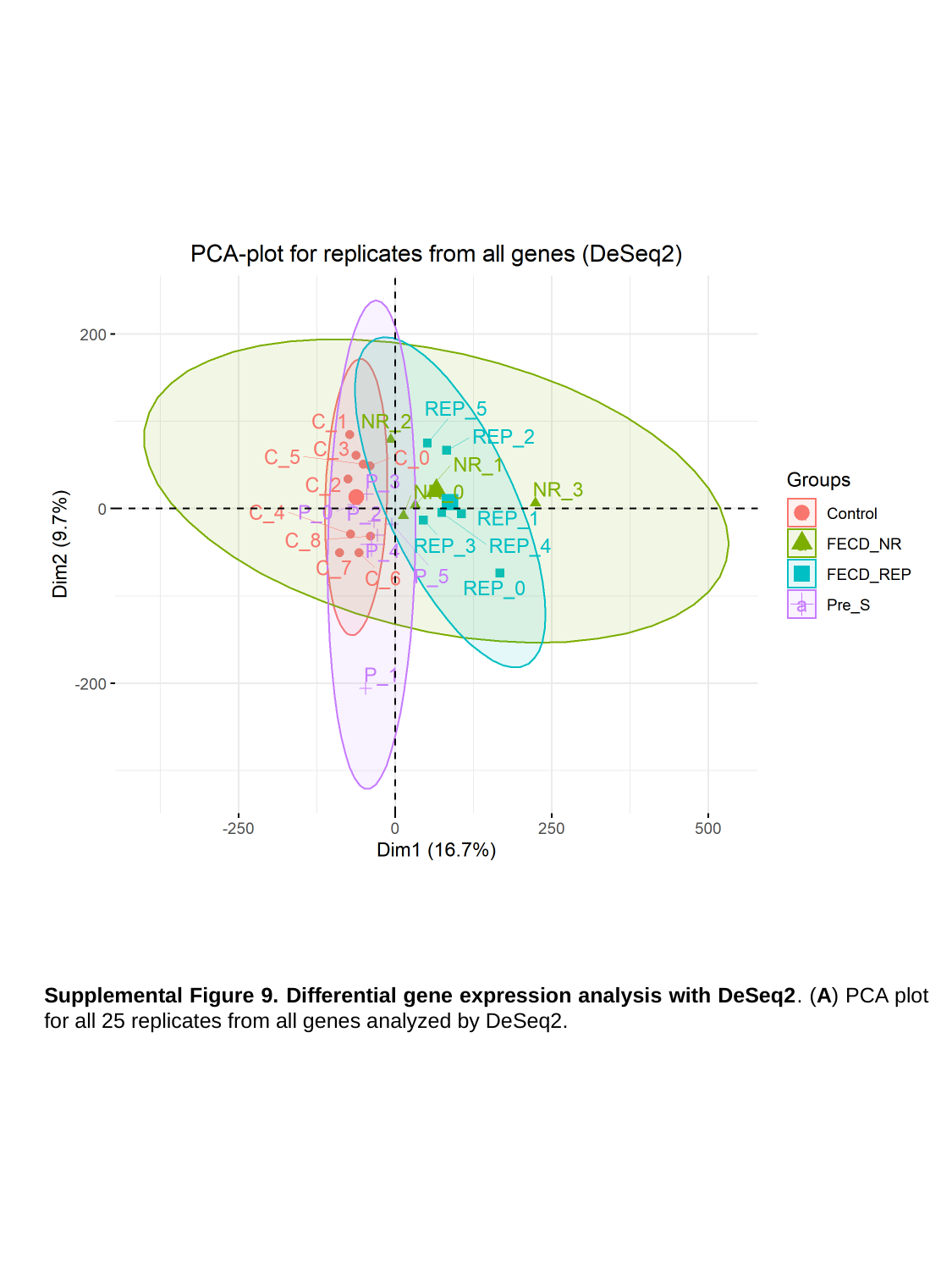

Supplemental Figure 9. Differential gene expression analysis with DeSeq2. (A) PCA plot for all 25 replicates from all genes analyzed by DeSeq2.

## Slide 13
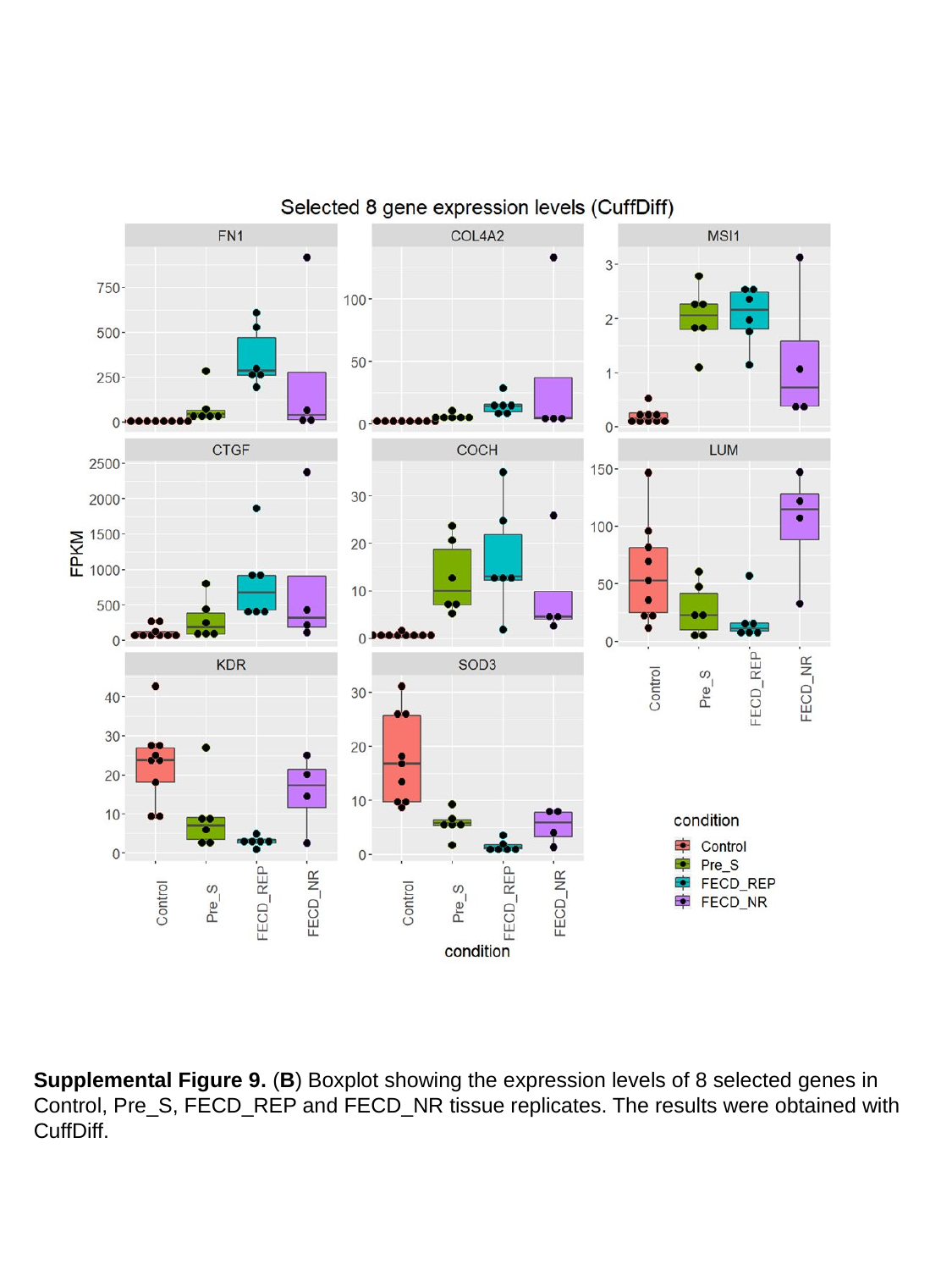

Supplemental Figure 9. (B) Boxplot showing the expression levels of 8 selected genes in Control, Pre_S, FECD_REP and FECD_NR tissue replicates. The results were obtained with CuffDiff.

## Slide 14
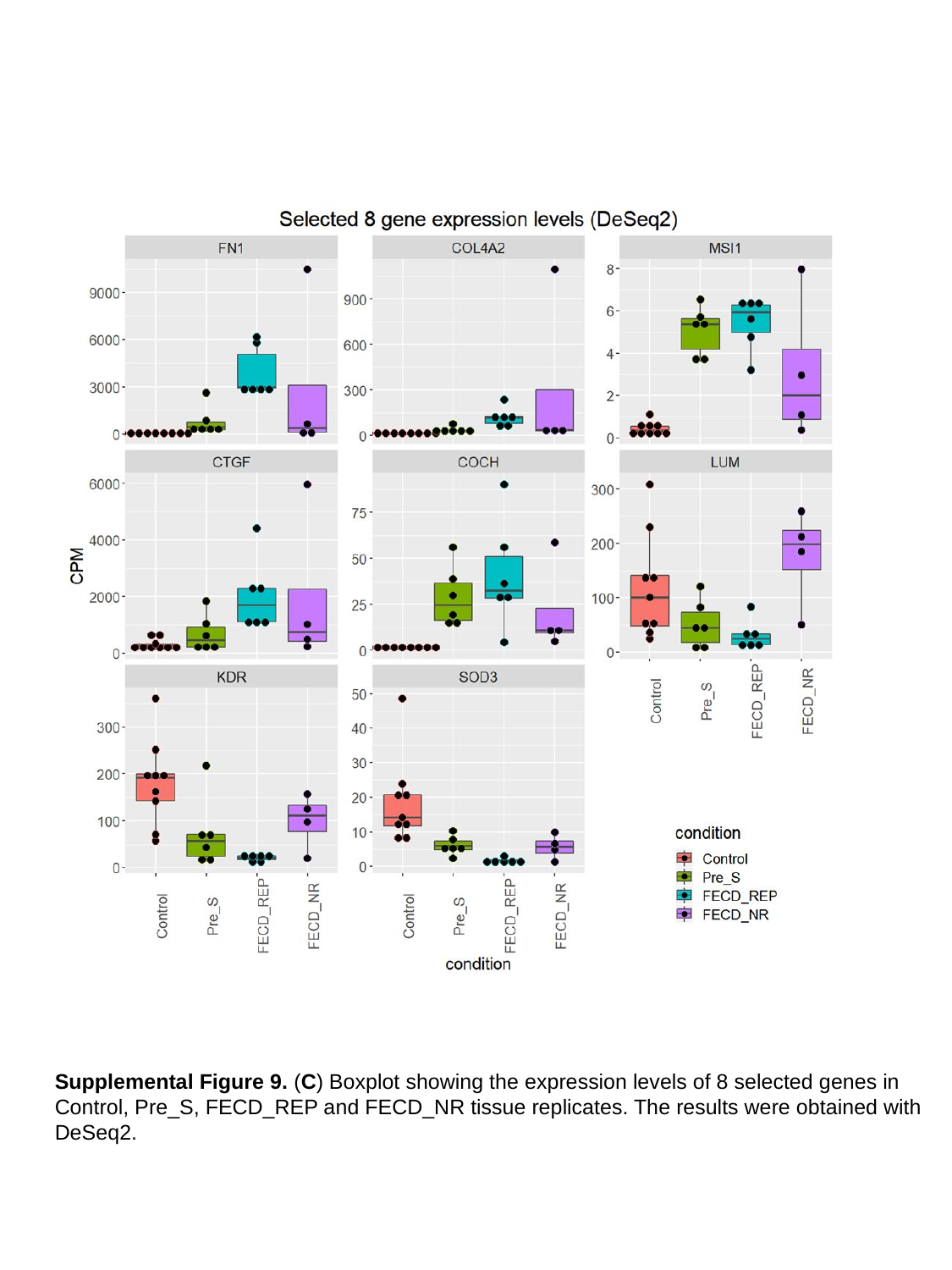

Supplemental Figure 9. (C) Boxplot showing the expression levels of 8 selected genes in Control, Pre_S, FECD_REP and FECD_NR tissue replicates. The results were obtained with DeSeq2.

## Slide 15
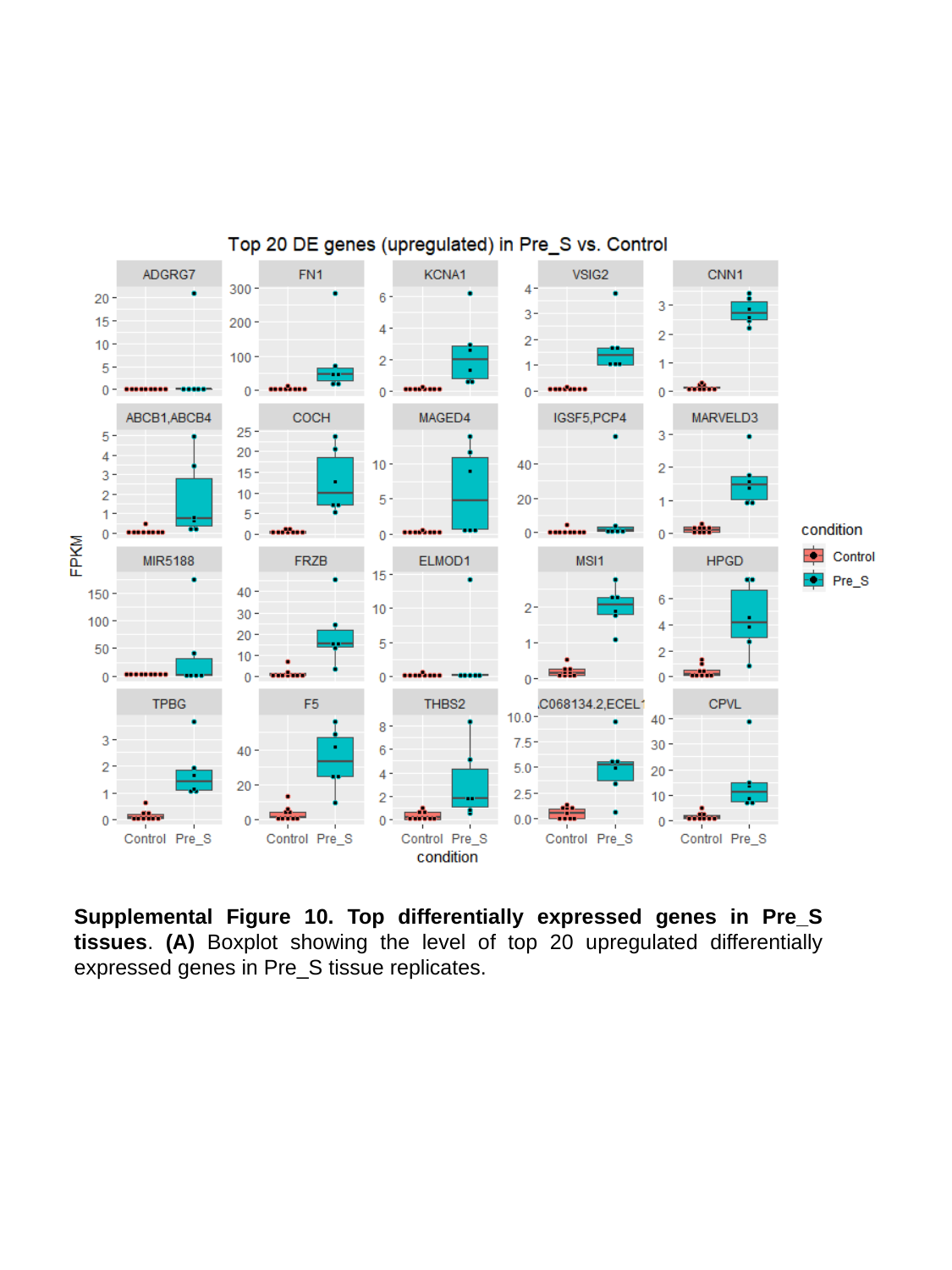

Supplemental Figure 10. Top differentially expressed genes in Pre_S tissues. (A) Boxplot showing the level of top 20 upregulated differentially expressed genes in Pre_S tissue replicates.

## Slide 16
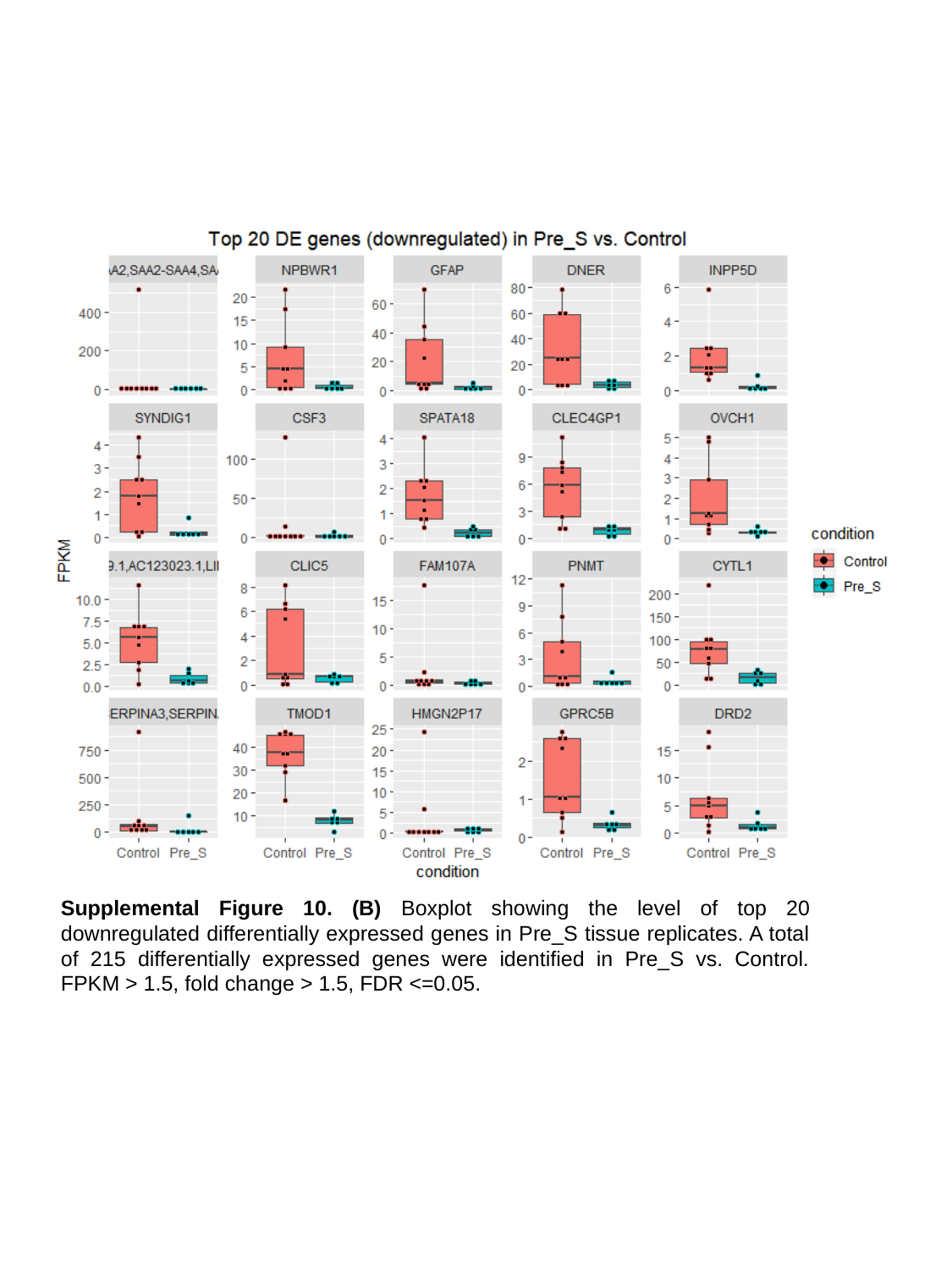

Supplemental Figure 10. (B) Boxplot showing the level of top 20 downregulated differentially expressed genes in Pre_S tissue replicates. A total of 215 differentially expressed genes were identified in Pre_S vs. Control. FPKM > 1.5, fold change > 1.5, FDR <=0.05.

## Slide 17
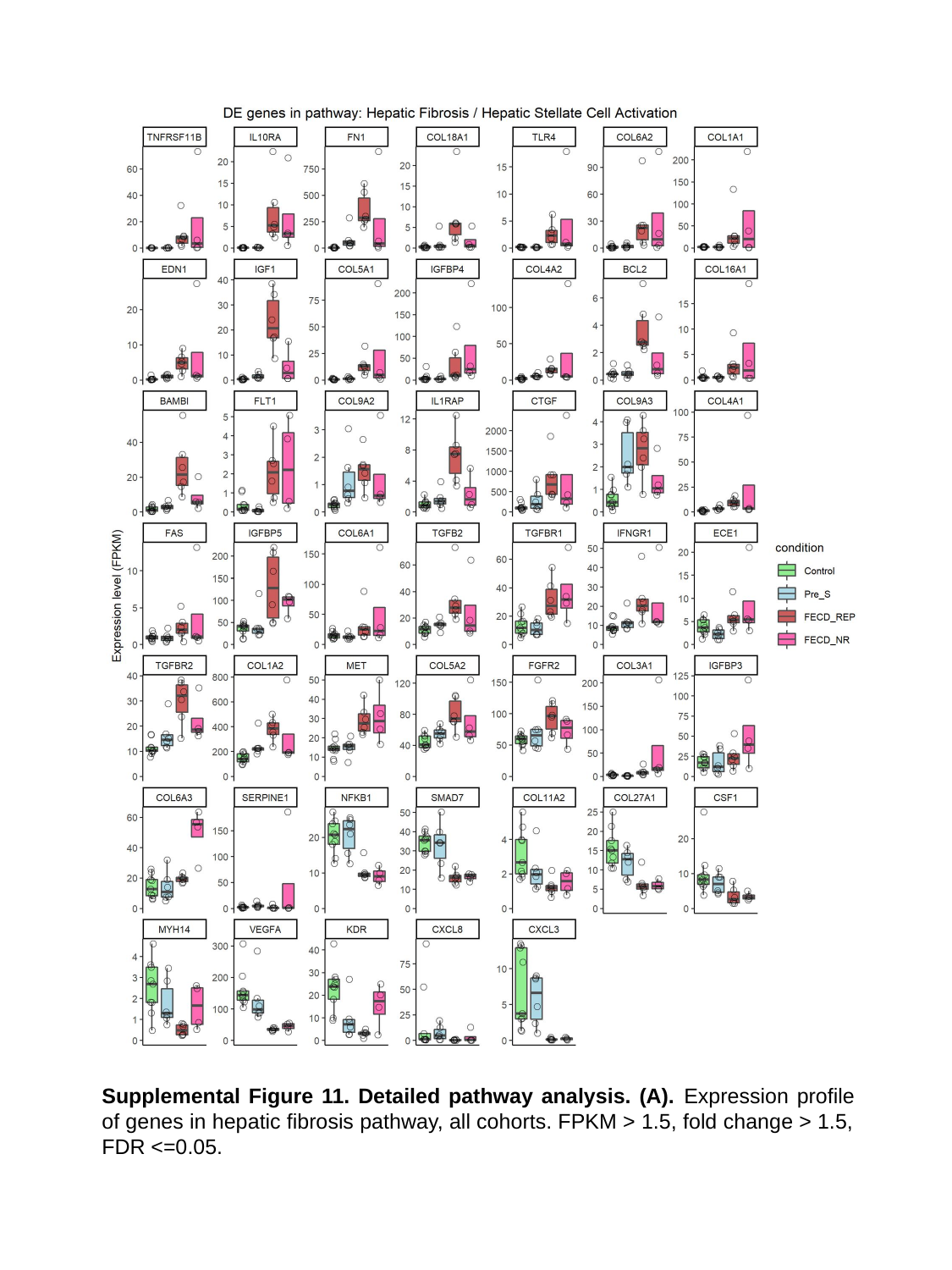

Supplemental Figure 11. Detailed pathway analysis. (A). Expression profile of genes in hepatic fibrosis pathway, all cohorts. FPKM > 1.5, fold change > 1.5, FDR <=0.05.

## Slide 18
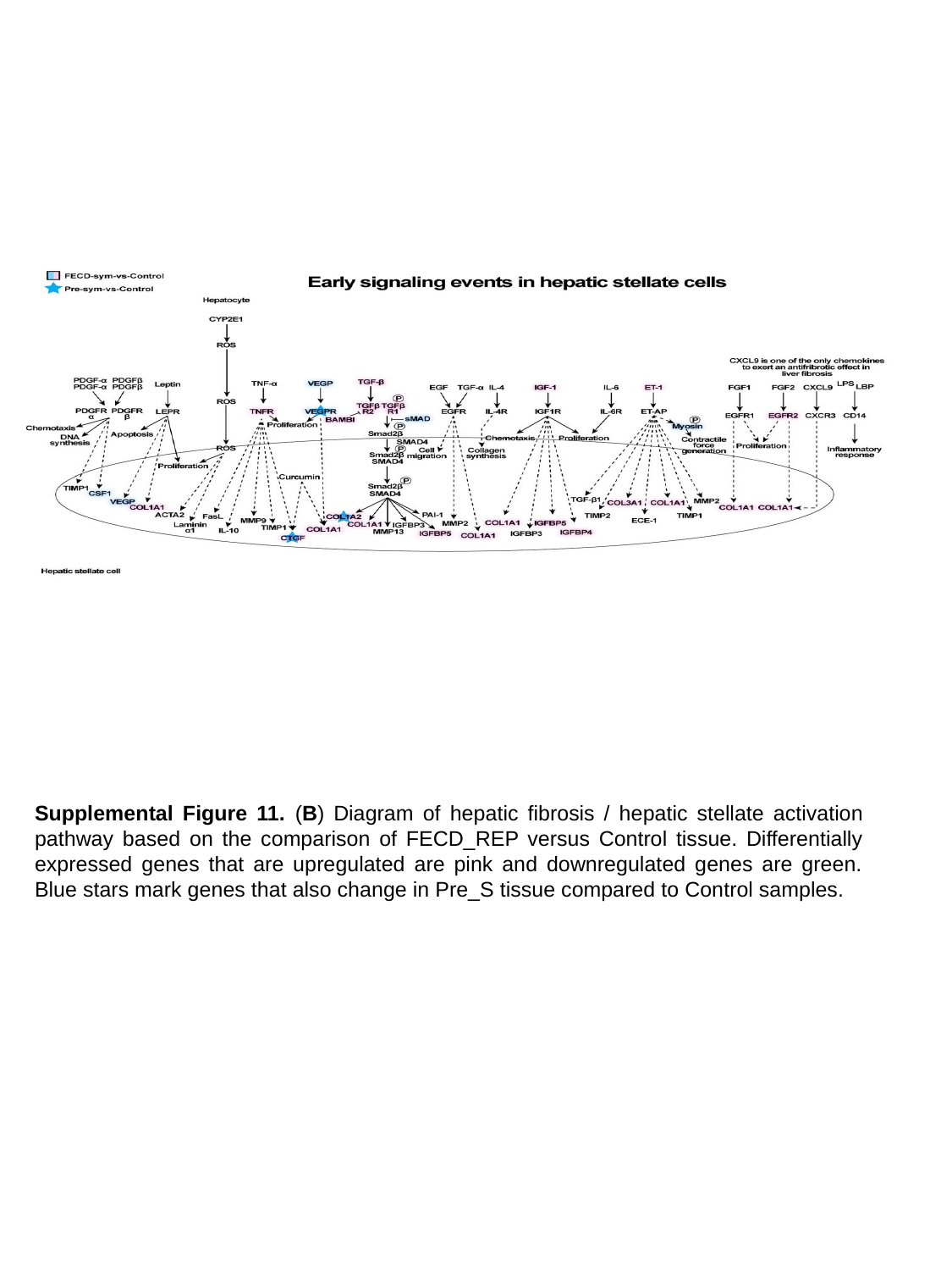

Supplemental Figure 11. (B) Diagram of hepatic fibrosis / hepatic stellate activation pathway based on the comparison of FECD_REP versus Control tissue. Differentially expressed genes that are upregulated are pink and downregulated genes are green. Blue stars mark genes that also change in Pre_S tissue compared to Control samples.

## Slide 19
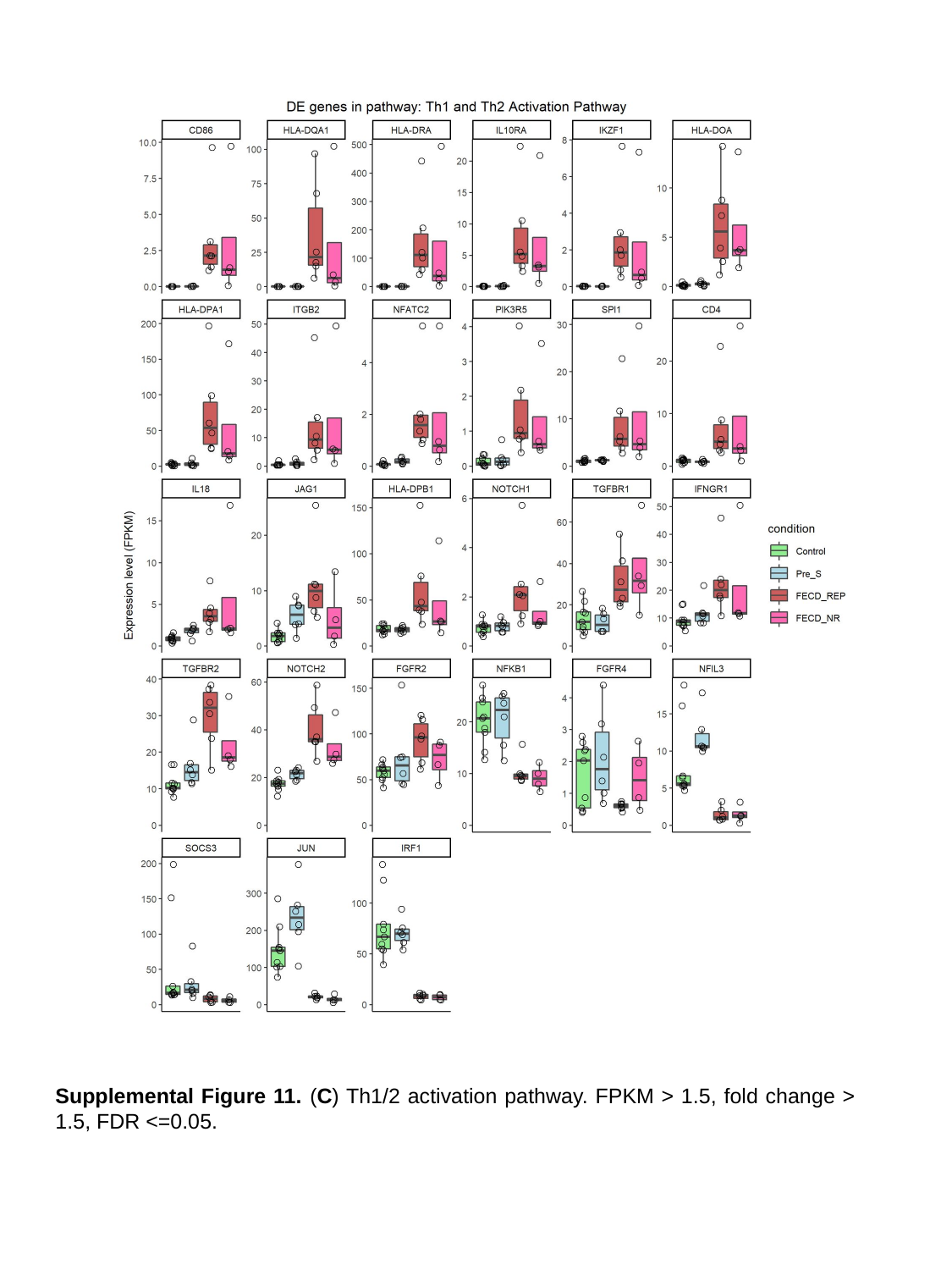

Supplemental Figure 11. (C) Th1/2 activation pathway. FPKM > 1.5, fold change > 1.5, FDR <=0.05.

## Slide 20
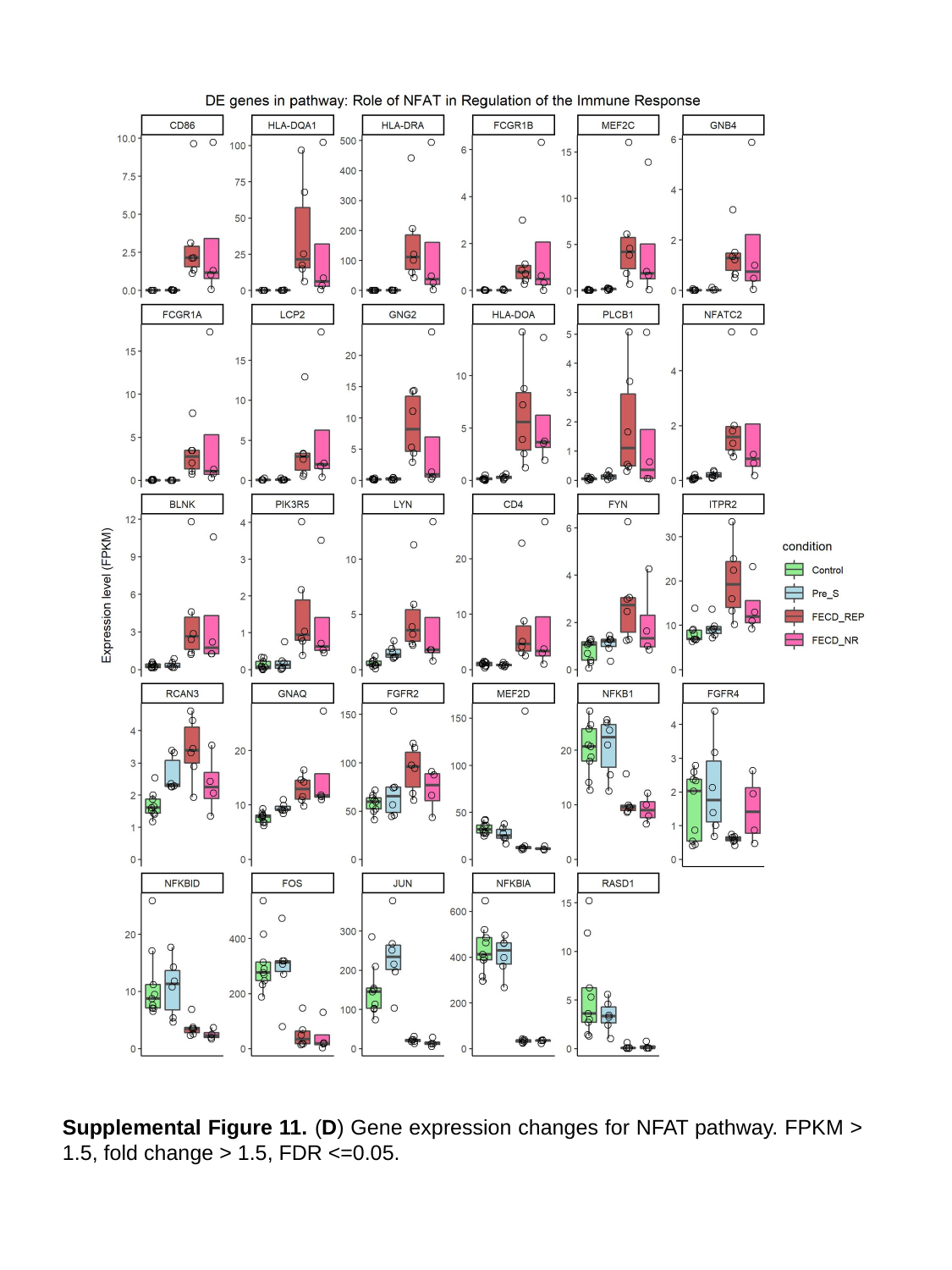

Supplemental Figure 11. (D) Gene expression changes for NFAT pathway. FPKM > 1.5, fold change > 1.5, FDR <=0.05.

## Slide 21
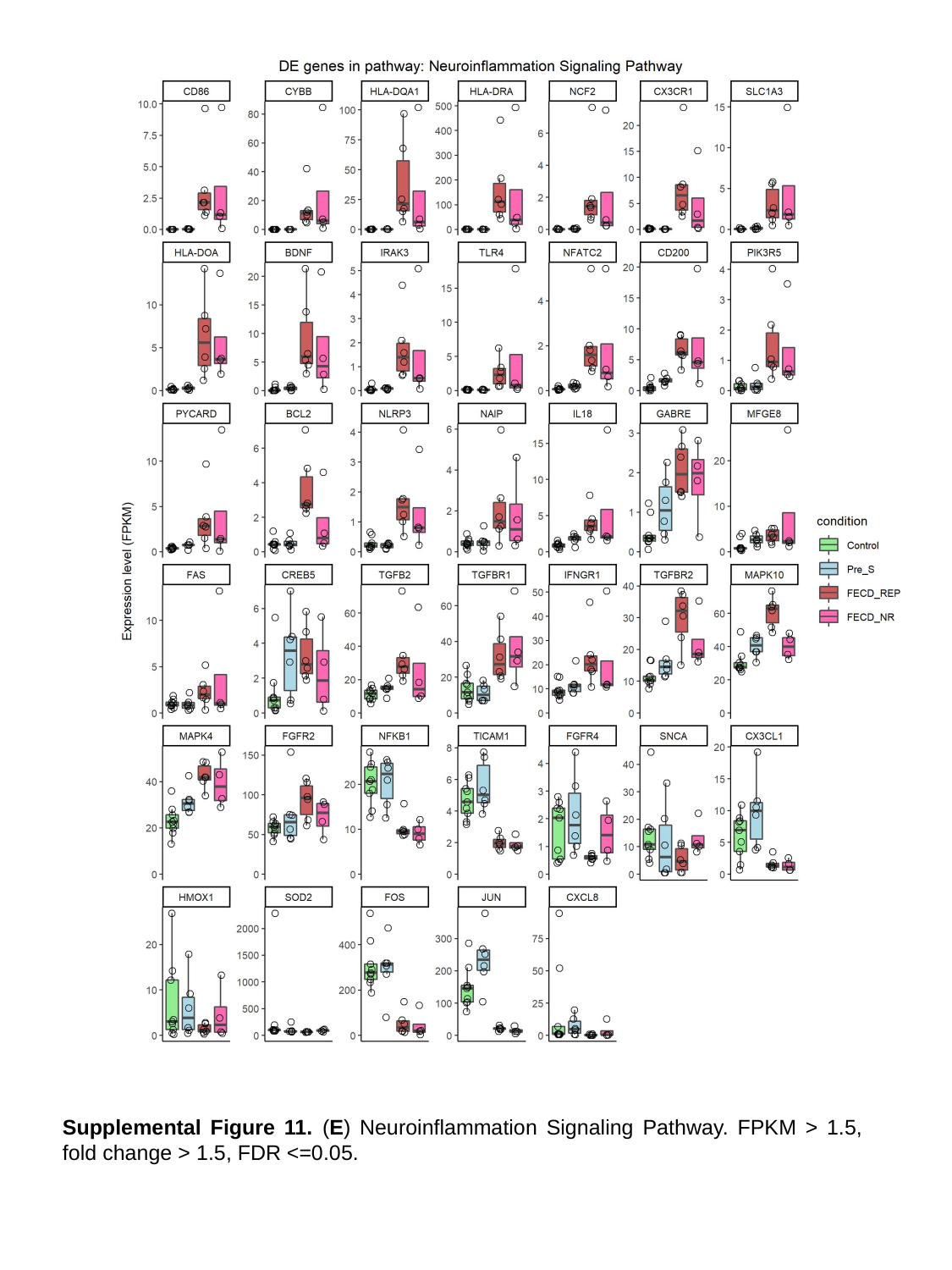

Supplemental Figure 11. (E) Neuroinflammation Signaling Pathway. FPKM > 1.5, fold change > 1.5, FDR <=0.05.

## Slide 22
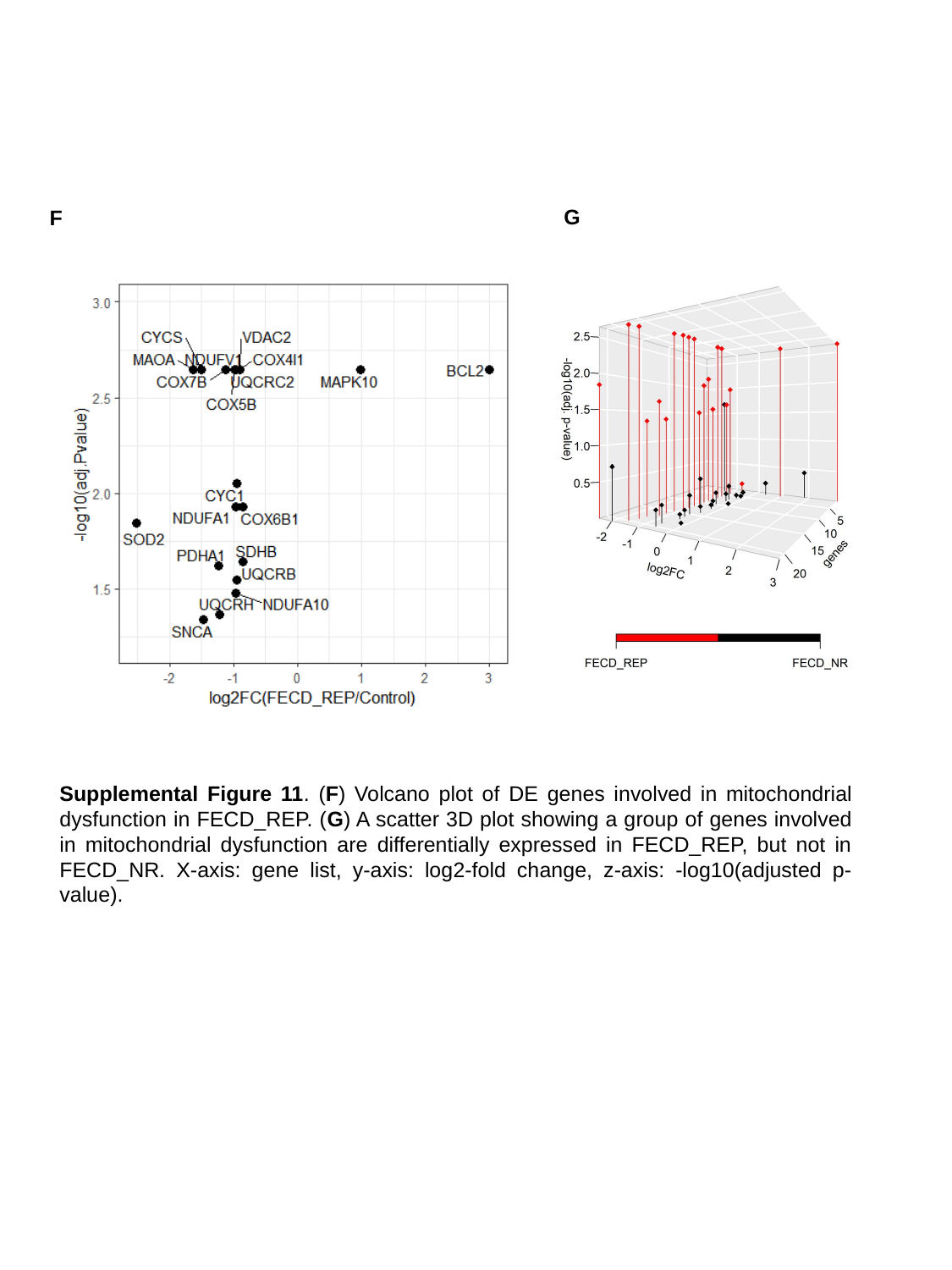

G
F
Supplemental Figure 11. (F) Volcano plot of DE genes involved in mitochondrial dysfunction in FECD_REP. (G) A scatter 3D plot showing a group of genes involved in mitochondrial dysfunction are differentially expressed in FECD_REP, but not in FECD_NR. X-axis: gene list, y-axis: log2-fold change, z-axis: -log10(adjusted p-value).

## Slide 23
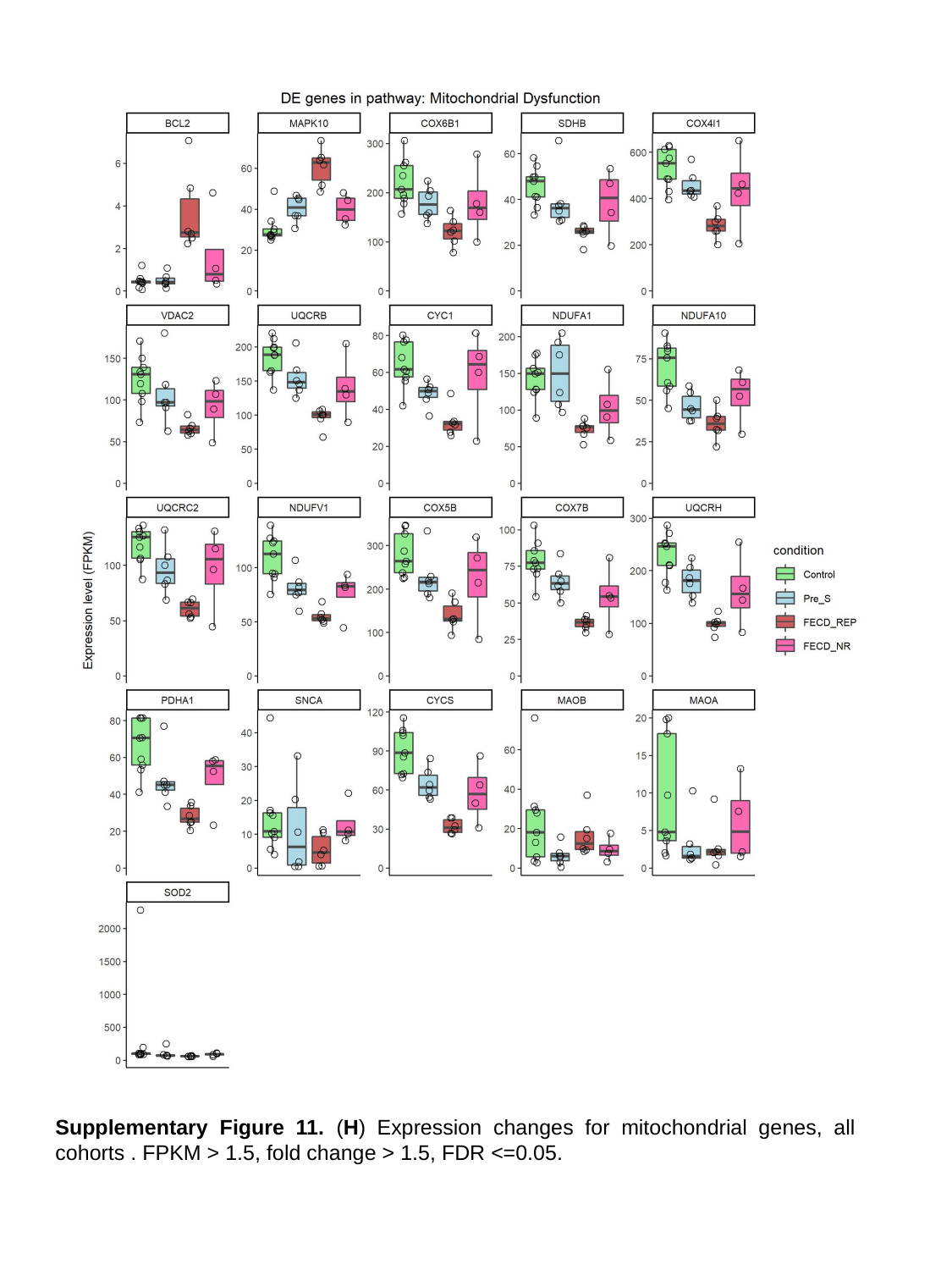

Supplementary Figure 11. (H) Expression changes for mitochondrial genes, all cohorts . FPKM > 1.5, fold change > 1.5, FDR <=0.05.

## Slide 24
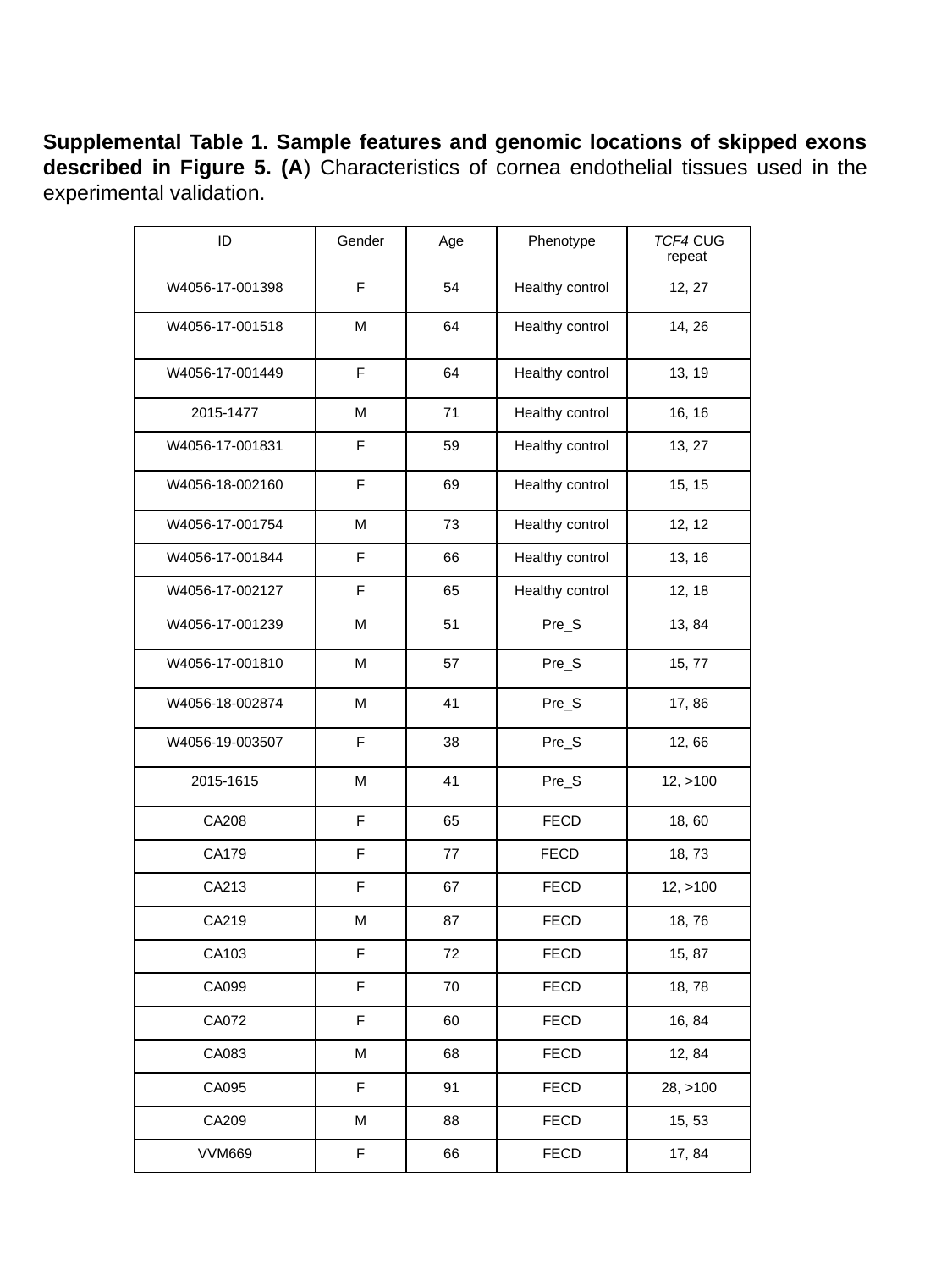

Supplemental Table 1. Sample features and genomic locations of skipped exons described in Figure 5. (A) Characteristics of cornea endothelial tissues used in the experimental validation.
| ID | Gender | Age | Phenotype | TCF4 CUG repeat |
| --- | --- | --- | --- | --- |
| W4056-17-001398 | F | 54 | Healthy control | 12, 27 |
| W4056-17-001518 | M | 64 | Healthy control | 14, 26 |
| W4056-17-001449 | F | 64 | Healthy control | 13, 19 |
| 2015-1477 | M | 71 | Healthy control | 16, 16 |
| W4056-17-001831 | F | 59 | Healthy control | 13, 27 |
| W4056-18-002160 | F | 69 | Healthy control | 15, 15 |
| W4056-17-001754 | M | 73 | Healthy control | 12, 12 |
| W4056-17-001844 | F | 66 | Healthy control | 13, 16 |
| W4056-17-002127 | F | 65 | Healthy control | 12, 18 |
| W4056-17-001239 | M | 51 | Pre\_S | 13, 84 |
| W4056-17-001810 | M | 57 | Pre\_S | 15, 77 |
| W4056-18-002874 | M | 41 | Pre\_S | 17, 86 |
| W4056-19-003507 | F | 38 | Pre\_S | 12, 66 |
| 2015-1615 | M | 41 | Pre\_S | 12, >100 |
| CA208 | F | 65 | FECD | 18, 60 |
| CA179 | F | 77 | FECD | 18, 73 |
| CA213 | F | 67 | FECD | 12, >100 |
| CA219 | M | 87 | FECD | 18, 76 |
| CA103 | F | 72 | FECD | 15, 87 |
| CA099 | F | 70 | FECD | 18, 78 |
| CA072 | F | 60 | FECD | 16, 84 |
| CA083 | M | 68 | FECD | 12, 84 |
| CA095 | F | 91 | FECD | 28, >100 |
| CA209 | M | 88 | FECD | 15, 53 |
| VVM669 | F | 66 | FECD | 17, 84 |

## Slide 25
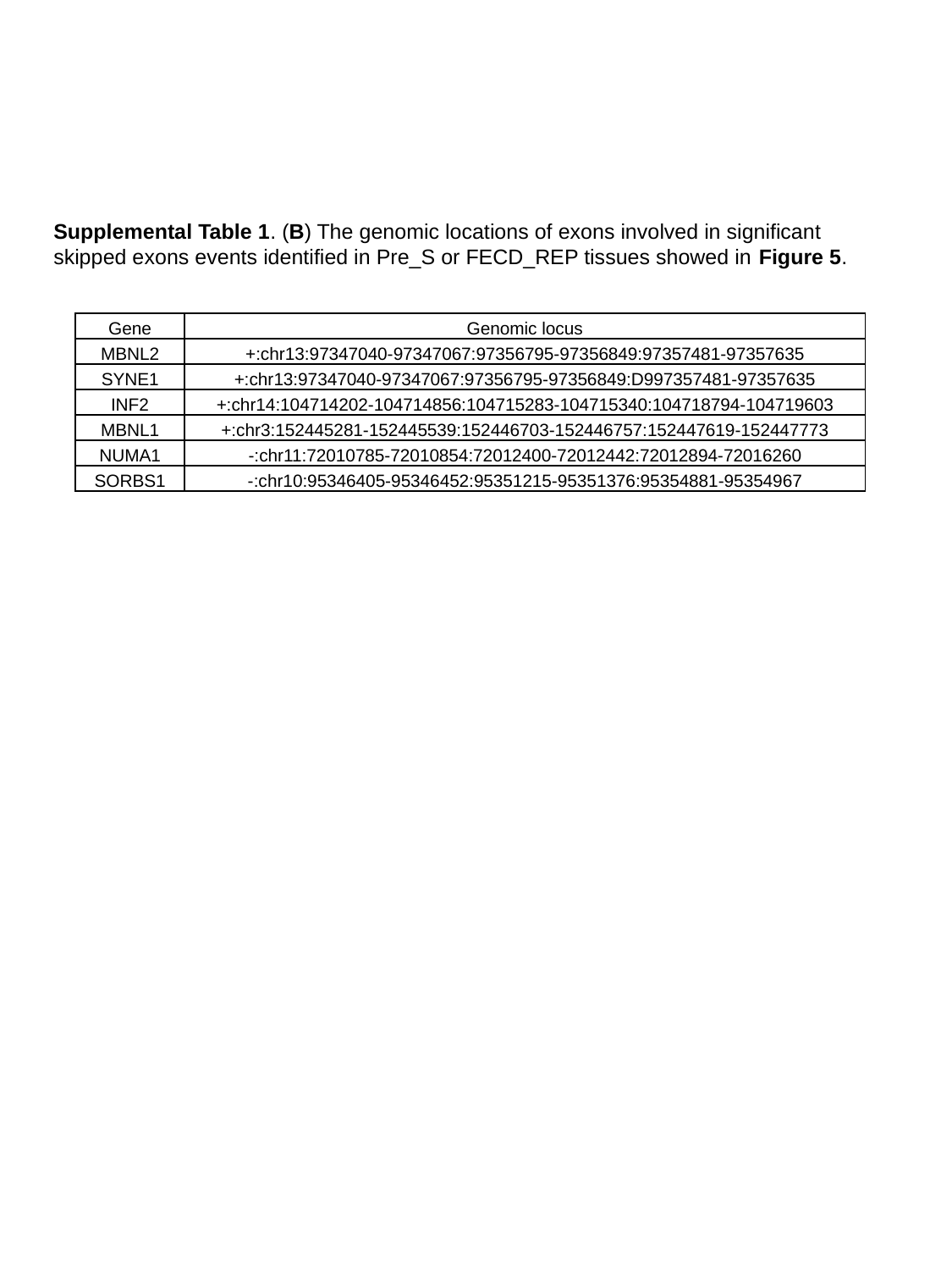

Supplemental Table 1. (B) The genomic locations of exons involved in significant skipped exons events identified in Pre_S or FECD_REP tissues showed in Figure 5.
| Gene | Genomic locus |
| --- | --- |
| MBNL2 | +:chr13:97347040-97347067:97356795-97356849:97357481-97357635 |
| SYNE1 | +:chr13:97347040-97347067:97356795-97356849:D997357481-97357635 |
| INF2 | +:chr14:104714202-104714856:104715283-104715340:104718794-104719603 |
| MBNL1 | +:chr3:152445281-152445539:152446703-152446757:152447619-152447773 |
| NUMA1 | -:chr11:72010785-72010854:72012400-72012442:72012894-72016260 |
| SORBS1 | -:chr10:95346405-95346452:95351215-95351376:95354881-95354967 |

## Slide 26
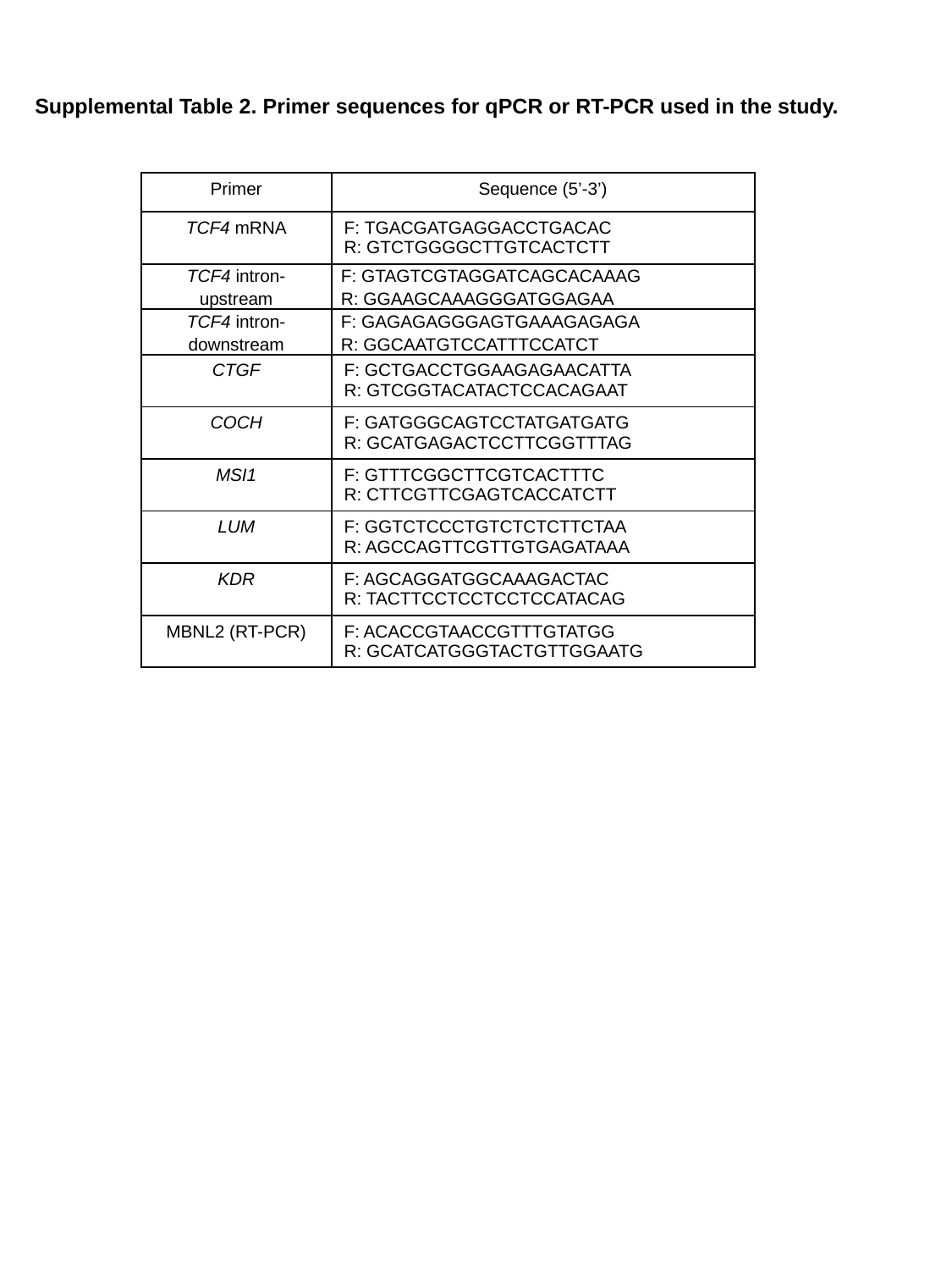

Supplemental Table 2. Primer sequences for qPCR or RT-PCR used in the study.
| Primer | Sequence (5’-3’) |
| --- | --- |
| TCF4 mRNA | F: TGACGATGAGGACCTGACAC R: GTCTGGGGCTTGTCACTCTT |
| TCF4 intron-upstream | F: GTAGTCGTAGGATCAGCACAAAG R: GGAAGCAAAGGGATGGAGAA |
| TCF4 intron-downstream | F: GAGAGAGGGAGTGAAAGAGAGA R: GGCAATGTCCATTTCCATCT |
| CTGF | F: GCTGACCTGGAAGAGAACATTA R: GTCGGTACATACTCCACAGAAT |
| COCH | F: GATGGGCAGTCCTATGATGATG R: GCATGAGACTCCTTCGGTTTAG |
| MSI1 | F: GTTTCGGCTTCGTCACTTTC R: CTTCGTTCGAGTCACCATCTT |
| LUM | F: GGTCTCCCTGTCTCTCTTCTAA R: AGCCAGTTCGTTGTGAGATAAA |
| KDR | F: AGCAGGATGGCAAAGACTAC R: TACTTCCTCCTCCTCCATACAG |
| MBNL2 (RT-PCR) | F: ACACCGTAACCGTTTGTATGG R: GCATCATGGGTACTGTTGGAATG |

## Slide 27
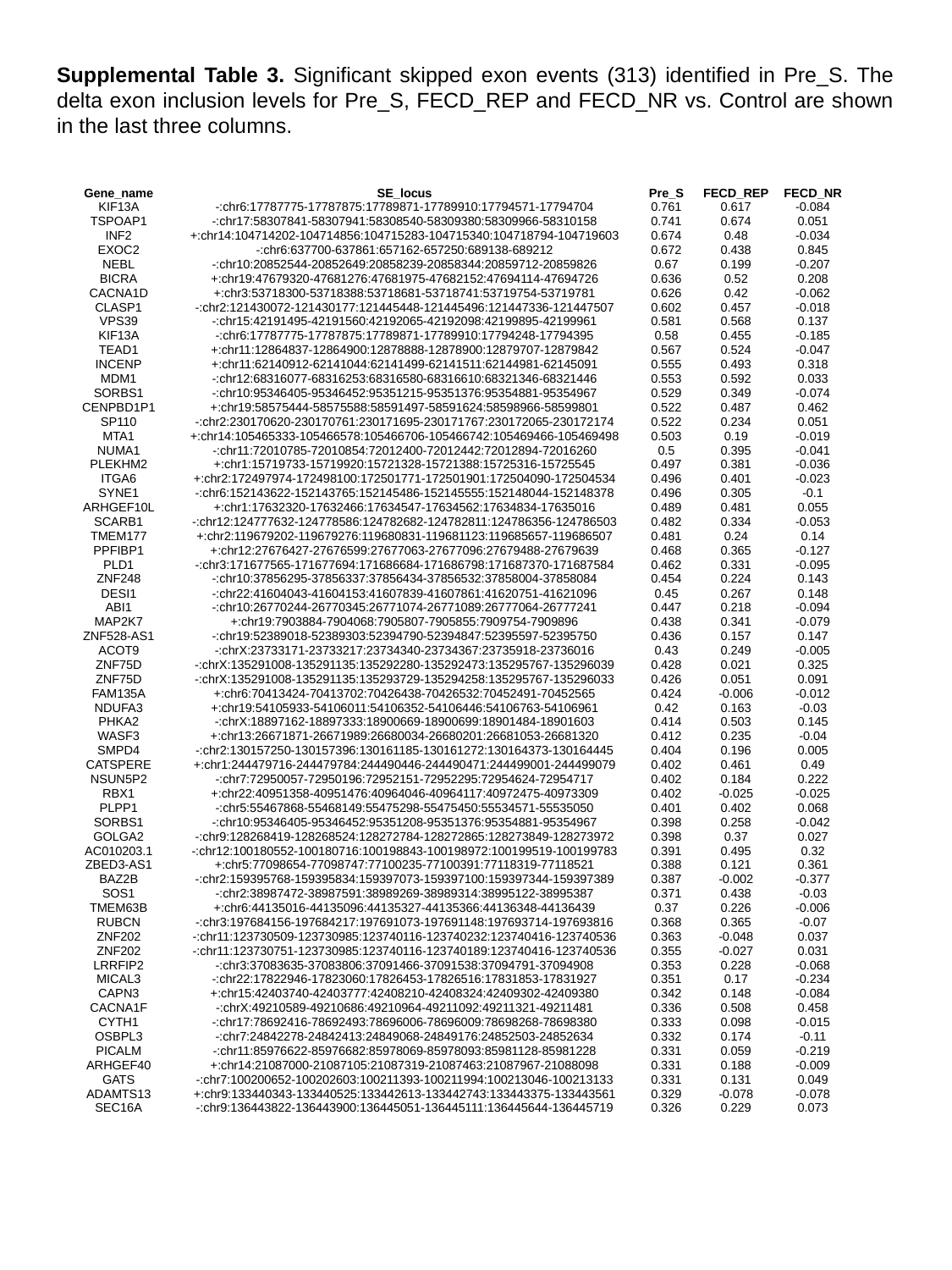

Supplemental Table 3. Significant skipped exon events (313) identified in Pre_S. The delta exon inclusion levels for Pre_S, FECD_REP and FECD_NR vs. Control are shown in the last three columns.
| Gene\_name | SE\_locus | Pre\_S | FECD\_REP | FECD\_NR |
| --- | --- | --- | --- | --- |
| KIF13A | -:chr6:17787775-17787875:17789871-17789910:17794571-17794704 | 0.761 | 0.617 | -0.084 |
| TSPOAP1 | -:chr17:58307841-58307941:58308540-58309380:58309966-58310158 | 0.741 | 0.674 | 0.051 |
| INF2 | +:chr14:104714202-104714856:104715283-104715340:104718794-104719603 | 0.674 | 0.48 | -0.034 |
| EXOC2 | -:chr6:637700-637861:657162-657250:689138-689212 | 0.672 | 0.438 | 0.845 |
| NEBL | -:chr10:20852544-20852649:20858239-20858344:20859712-20859826 | 0.67 | 0.199 | -0.207 |
| BICRA | +:chr19:47679320-47681276:47681975-47682152:47694114-47694726 | 0.636 | 0.52 | 0.208 |
| CACNA1D | +:chr3:53718300-53718388:53718681-53718741:53719754-53719781 | 0.626 | 0.42 | -0.062 |
| CLASP1 | -:chr2:121430072-121430177:121445448-121445496:121447336-121447507 | 0.602 | 0.457 | -0.018 |
| VPS39 | -:chr15:42191495-42191560:42192065-42192098:42199895-42199961 | 0.581 | 0.568 | 0.137 |
| KIF13A | -:chr6:17787775-17787875:17789871-17789910:17794248-17794395 | 0.58 | 0.455 | -0.185 |
| TEAD1 | +:chr11:12864837-12864900:12878888-12878900:12879707-12879842 | 0.567 | 0.524 | -0.047 |
| INCENP | +:chr11:62140912-62141044:62141499-62141511:62144981-62145091 | 0.555 | 0.493 | 0.318 |
| MDM1 | -:chr12:68316077-68316253:68316580-68316610:68321346-68321446 | 0.553 | 0.592 | 0.033 |
| SORBS1 | -:chr10:95346405-95346452:95351215-95351376:95354881-95354967 | 0.529 | 0.349 | -0.074 |
| CENPBD1P1 | +:chr19:58575444-58575588:58591497-58591624:58598966-58599801 | 0.522 | 0.487 | 0.462 |
| SP110 | -:chr2:230170620-230170761:230171695-230171767:230172065-230172174 | 0.522 | 0.234 | 0.051 |
| MTA1 | +:chr14:105465333-105466578:105466706-105466742:105469466-105469498 | 0.503 | 0.19 | -0.019 |
| NUMA1 | -:chr11:72010785-72010854:72012400-72012442:72012894-72016260 | 0.5 | 0.395 | -0.041 |
| PLEKHM2 | +:chr1:15719733-15719920:15721328-15721388:15725316-15725545 | 0.497 | 0.381 | -0.036 |
| ITGA6 | +:chr2:172497974-172498100:172501771-172501901:172504090-172504534 | 0.496 | 0.401 | -0.023 |
| SYNE1 | -:chr6:152143622-152143765:152145486-152145555:152148044-152148378 | 0.496 | 0.305 | -0.1 |
| ARHGEF10L | +:chr1:17632320-17632466:17634547-17634562:17634834-17635016 | 0.489 | 0.481 | 0.055 |
| SCARB1 | -:chr12:124777632-124778586:124782682-124782811:124786356-124786503 | 0.482 | 0.334 | -0.053 |
| TMEM177 | +:chr2:119679202-119679276:119680831-119681123:119685657-119686507 | 0.481 | 0.24 | 0.14 |
| PPFIBP1 | +:chr12:27676427-27676599:27677063-27677096:27679488-27679639 | 0.468 | 0.365 | -0.127 |
| PLD1 | -:chr3:171677565-171677694:171686684-171686798:171687370-171687584 | 0.462 | 0.331 | -0.095 |
| ZNF248 | -:chr10:37856295-37856337:37856434-37856532:37858004-37858084 | 0.454 | 0.224 | 0.143 |
| DESI1 | -:chr22:41604043-41604153:41607839-41607861:41620751-41621096 | 0.45 | 0.267 | 0.148 |
| ABI1 | -:chr10:26770244-26770345:26771074-26771089:26777064-26777241 | 0.447 | 0.218 | -0.094 |
| MAP2K7 | +:chr19:7903884-7904068:7905807-7905855:7909754-7909896 | 0.438 | 0.341 | -0.079 |
| ZNF528-AS1 | -:chr19:52389018-52389303:52394790-52394847:52395597-52395750 | 0.436 | 0.157 | 0.147 |
| ACOT9 | -:chrX:23733171-23733217:23734340-23734367:23735918-23736016 | 0.43 | 0.249 | -0.005 |
| ZNF75D | -:chrX:135291008-135291135:135292280-135292473:135295767-135296039 | 0.428 | 0.021 | 0.325 |
| ZNF75D | -:chrX:135291008-135291135:135293729-135294258:135295767-135296033 | 0.426 | 0.051 | 0.091 |
| FAM135A | +:chr6:70413424-70413702:70426438-70426532:70452491-70452565 | 0.424 | -0.006 | -0.012 |
| NDUFA3 | +:chr19:54105933-54106011:54106352-54106446:54106763-54106961 | 0.42 | 0.163 | -0.03 |
| PHKA2 | -:chrX:18897162-18897333:18900669-18900699:18901484-18901603 | 0.414 | 0.503 | 0.145 |
| WASF3 | +:chr13:26671871-26671989:26680034-26680201:26681053-26681320 | 0.412 | 0.235 | -0.04 |
| SMPD4 | -:chr2:130157250-130157396:130161185-130161272:130164373-130164445 | 0.404 | 0.196 | 0.005 |
| CATSPERE | +:chr1:244479716-244479784:244490446-244490471:244499001-244499079 | 0.402 | 0.461 | 0.49 |
| NSUN5P2 | -:chr7:72950057-72950196:72952151-72952295:72954624-72954717 | 0.402 | 0.184 | 0.222 |
| RBX1 | +:chr22:40951358-40951476:40964046-40964117:40972475-40973309 | 0.402 | -0.025 | -0.025 |
| PLPP1 | -:chr5:55467868-55468149:55475298-55475450:55534571-55535050 | 0.401 | 0.402 | 0.068 |
| SORBS1 | -:chr10:95346405-95346452:95351208-95351376:95354881-95354967 | 0.398 | 0.258 | -0.042 |
| GOLGA2 | -:chr9:128268419-128268524:128272784-128272865:128273849-128273972 | 0.398 | 0.37 | 0.027 |
| AC010203.1 | -:chr12:100180552-100180716:100198843-100198972:100199519-100199783 | 0.391 | 0.495 | 0.32 |
| ZBED3-AS1 | +:chr5:77098654-77098747:77100235-77100391:77118319-77118521 | 0.388 | 0.121 | 0.361 |
| BAZ2B | -:chr2:159395768-159395834:159397073-159397100:159397344-159397389 | 0.387 | -0.002 | -0.377 |
| SOS1 | -:chr2:38987472-38987591:38989269-38989314:38995122-38995387 | 0.371 | 0.438 | -0.03 |
| TMEM63B | +:chr6:44135016-44135096:44135327-44135366:44136348-44136439 | 0.37 | 0.226 | -0.006 |
| RUBCN | -:chr3:197684156-197684217:197691073-197691148:197693714-197693816 | 0.368 | 0.365 | -0.07 |
| ZNF202 | -:chr11:123730509-123730985:123740116-123740232:123740416-123740536 | 0.363 | -0.048 | 0.037 |
| ZNF202 | -:chr11:123730751-123730985:123740116-123740189:123740416-123740536 | 0.355 | -0.027 | 0.031 |
| LRRFIP2 | -:chr3:37083635-37083806:37091466-37091538:37094791-37094908 | 0.353 | 0.228 | -0.068 |
| MICAL3 | -:chr22:17822946-17823060:17826453-17826516:17831853-17831927 | 0.351 | 0.17 | -0.234 |
| CAPN3 | +:chr15:42403740-42403777:42408210-42408324:42409302-42409380 | 0.342 | 0.148 | -0.084 |
| CACNA1F | -:chrX:49210589-49210686:49210964-49211092:49211321-49211481 | 0.336 | 0.508 | 0.458 |
| CYTH1 | -:chr17:78692416-78692493:78696006-78696009:78698268-78698380 | 0.333 | 0.098 | -0.015 |
| OSBPL3 | -:chr7:24842278-24842413:24849068-24849176:24852503-24852634 | 0.332 | 0.174 | -0.11 |
| PICALM | -:chr11:85976622-85976682:85978069-85978093:85981128-85981228 | 0.331 | 0.059 | -0.219 |
| ARHGEF40 | +:chr14:21087000-21087105:21087319-21087463:21087967-21088098 | 0.331 | 0.188 | -0.009 |
| GATS | -:chr7:100200652-100202603:100211393-100211994:100213046-100213133 | 0.331 | 0.131 | 0.049 |
| ADAMTS13 | +:chr9:133440343-133440525:133442613-133442743:133443375-133443561 | 0.329 | -0.078 | -0.078 |
| SEC16A | -:chr9:136443822-136443900:136445051-136445111:136445644-136445719 | 0.326 | 0.229 | 0.073 |

## Slide 28
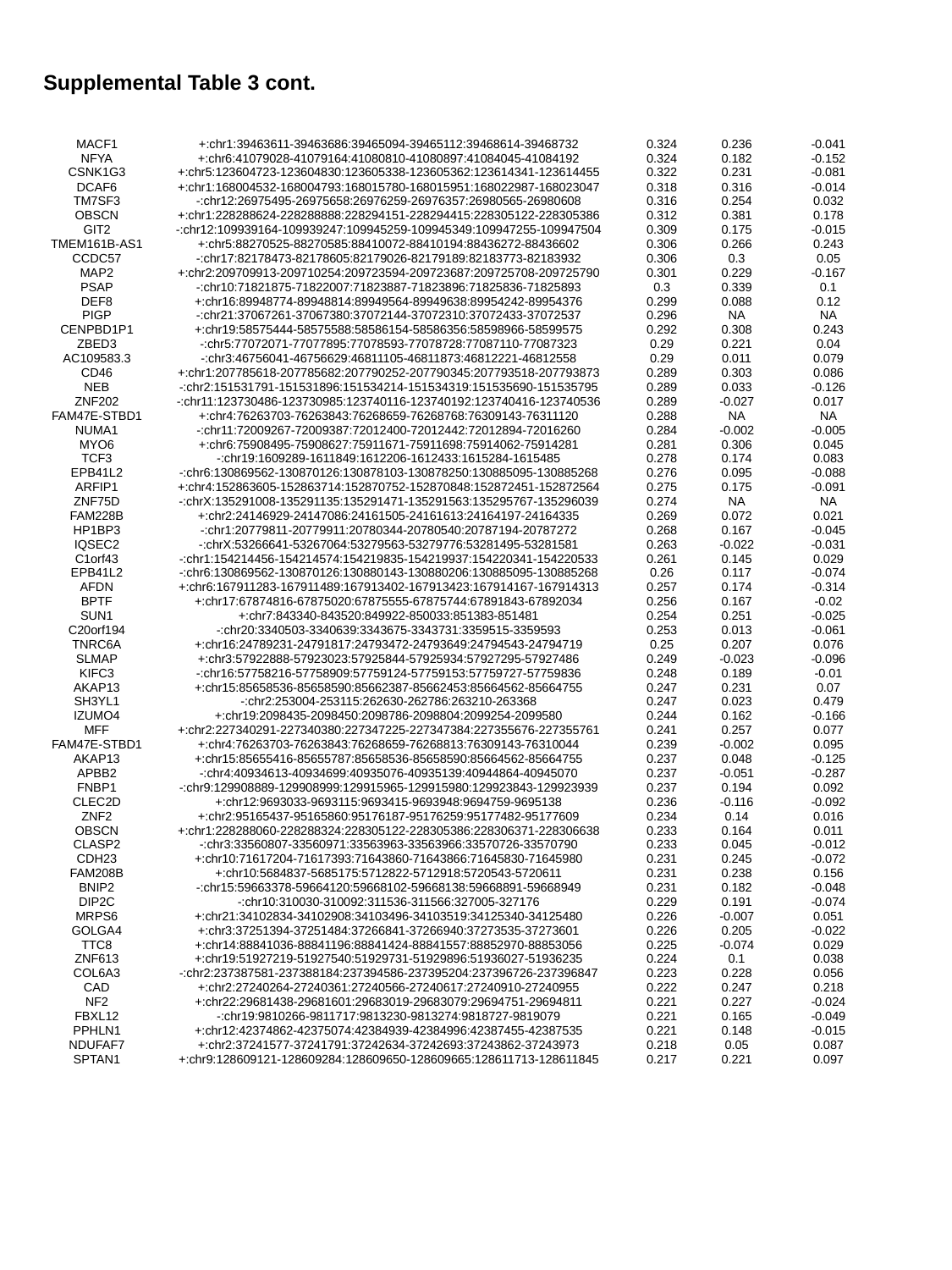

Supplemental Table 3 cont.
| MACF1 | +:chr1:39463611-39463686:39465094-39465112:39468614-39468732 | 0.324 | 0.236 | -0.041 |
| --- | --- | --- | --- | --- |
| NFYA | +:chr6:41079028-41079164:41080810-41080897:41084045-41084192 | 0.324 | 0.182 | -0.152 |
| CSNK1G3 | +:chr5:123604723-123604830:123605338-123605362:123614341-123614455 | 0.322 | 0.231 | -0.081 |
| DCAF6 | +:chr1:168004532-168004793:168015780-168015951:168022987-168023047 | 0.318 | 0.316 | -0.014 |
| TM7SF3 | -:chr12:26975495-26975658:26976259-26976357:26980565-26980608 | 0.316 | 0.254 | 0.032 |
| OBSCN | +:chr1:228288624-228288888:228294151-228294415:228305122-228305386 | 0.312 | 0.381 | 0.178 |
| GIT2 | -:chr12:109939164-109939247:109945259-109945349:109947255-109947504 | 0.309 | 0.175 | -0.015 |
| TMEM161B-AS1 | +:chr5:88270525-88270585:88410072-88410194:88436272-88436602 | 0.306 | 0.266 | 0.243 |
| CCDC57 | -:chr17:82178473-82178605:82179026-82179189:82183773-82183932 | 0.306 | 0.3 | 0.05 |
| MAP2 | +:chr2:209709913-209710254:209723594-209723687:209725708-209725790 | 0.301 | 0.229 | -0.167 |
| PSAP | -:chr10:71821875-71822007:71823887-71823896:71825836-71825893 | 0.3 | 0.339 | 0.1 |
| DEF8 | +:chr16:89948774-89948814:89949564-89949638:89954242-89954376 | 0.299 | 0.088 | 0.12 |
| PIGP | -:chr21:37067261-37067380:37072144-37072310:37072433-37072537 | 0.296 | NA | NA |
| CENPBD1P1 | +:chr19:58575444-58575588:58586154-58586356:58598966-58599575 | 0.292 | 0.308 | 0.243 |
| ZBED3 | -:chr5:77072071-77077895:77078593-77078728:77087110-77087323 | 0.29 | 0.221 | 0.04 |
| AC109583.3 | -:chr3:46756041-46756629:46811105-46811873:46812221-46812558 | 0.29 | 0.011 | 0.079 |
| CD46 | +:chr1:207785618-207785682:207790252-207790345:207793518-207793873 | 0.289 | 0.303 | 0.086 |
| NEB | -:chr2:151531791-151531896:151534214-151534319:151535690-151535795 | 0.289 | 0.033 | -0.126 |
| ZNF202 | -:chr11:123730486-123730985:123740116-123740192:123740416-123740536 | 0.289 | -0.027 | 0.017 |
| FAM47E-STBD1 | +:chr4:76263703-76263843:76268659-76268768:76309143-76311120 | 0.288 | NA | NA |
| NUMA1 | -:chr11:72009267-72009387:72012400-72012442:72012894-72016260 | 0.284 | -0.002 | -0.005 |
| MYO6 | +:chr6:75908495-75908627:75911671-75911698:75914062-75914281 | 0.281 | 0.306 | 0.045 |
| TCF3 | -:chr19:1609289-1611849:1612206-1612433:1615284-1615485 | 0.278 | 0.174 | 0.083 |
| EPB41L2 | -:chr6:130869562-130870126:130878103-130878250:130885095-130885268 | 0.276 | 0.095 | -0.088 |
| ARFIP1 | +:chr4:152863605-152863714:152870752-152870848:152872451-152872564 | 0.275 | 0.175 | -0.091 |
| ZNF75D | -:chrX:135291008-135291135:135291471-135291563:135295767-135296039 | 0.274 | NA | NA |
| FAM228B | +:chr2:24146929-24147086:24161505-24161613:24164197-24164335 | 0.269 | 0.072 | 0.021 |
| HP1BP3 | -:chr1:20779811-20779911:20780344-20780540:20787194-20787272 | 0.268 | 0.167 | -0.045 |
| IQSEC2 | -:chrX:53266641-53267064:53279563-53279776:53281495-53281581 | 0.263 | -0.022 | -0.031 |
| C1orf43 | -:chr1:154214456-154214574:154219835-154219937:154220341-154220533 | 0.261 | 0.145 | 0.029 |
| EPB41L2 | -:chr6:130869562-130870126:130880143-130880206:130885095-130885268 | 0.26 | 0.117 | -0.074 |
| AFDN | +:chr6:167911283-167911489:167913402-167913423:167914167-167914313 | 0.257 | 0.174 | -0.314 |
| BPTF | +:chr17:67874816-67875020:67875555-67875744:67891843-67892034 | 0.256 | 0.167 | -0.02 |
| SUN1 | +:chr7:843340-843520:849922-850033:851383-851481 | 0.254 | 0.251 | -0.025 |
| C20orf194 | -:chr20:3340503-3340639:3343675-3343731:3359515-3359593 | 0.253 | 0.013 | -0.061 |
| TNRC6A | +:chr16:24789231-24791817:24793472-24793649:24794543-24794719 | 0.25 | 0.207 | 0.076 |
| SLMAP | +:chr3:57922888-57923023:57925844-57925934:57927295-57927486 | 0.249 | -0.023 | -0.096 |
| KIFC3 | -:chr16:57758216-57758909:57759124-57759153:57759727-57759836 | 0.248 | 0.189 | -0.01 |
| AKAP13 | +:chr15:85658536-85658590:85662387-85662453:85664562-85664755 | 0.247 | 0.231 | 0.07 |
| SH3YL1 | -:chr2:253004-253115:262630-262786:263210-263368 | 0.247 | 0.023 | 0.479 |
| IZUMO4 | +:chr19:2098435-2098450:2098786-2098804:2099254-2099580 | 0.244 | 0.162 | -0.166 |
| MFF | +:chr2:227340291-227340380:227347225-227347384:227355676-227355761 | 0.241 | 0.257 | 0.077 |
| FAM47E-STBD1 | +:chr4:76263703-76263843:76268659-76268813:76309143-76310044 | 0.239 | -0.002 | 0.095 |
| AKAP13 | +:chr15:85655416-85655787:85658536-85658590:85664562-85664755 | 0.237 | 0.048 | -0.125 |
| APBB2 | -:chr4:40934613-40934699:40935076-40935139:40944864-40945070 | 0.237 | -0.051 | -0.287 |
| FNBP1 | -:chr9:129908889-129908999:129915965-129915980:129923843-129923939 | 0.237 | 0.194 | 0.092 |
| CLEC2D | +:chr12:9693033-9693115:9693415-9693948:9694759-9695138 | 0.236 | -0.116 | -0.092 |
| ZNF2 | +:chr2:95165437-95165860:95176187-95176259:95177482-95177609 | 0.234 | 0.14 | 0.016 |
| OBSCN | +:chr1:228288060-228288324:228305122-228305386:228306371-228306638 | 0.233 | 0.164 | 0.011 |
| CLASP2 | -:chr3:33560807-33560971:33563963-33563966:33570726-33570790 | 0.233 | 0.045 | -0.012 |
| CDH23 | +:chr10:71617204-71617393:71643860-71643866:71645830-71645980 | 0.231 | 0.245 | -0.072 |
| FAM208B | +:chr10:5684837-5685175:5712822-5712918:5720543-5720611 | 0.231 | 0.238 | 0.156 |
| BNIP2 | -:chr15:59663378-59664120:59668102-59668138:59668891-59668949 | 0.231 | 0.182 | -0.048 |
| DIP2C | -:chr10:310030-310092:311536-311566:327005-327176 | 0.229 | 0.191 | -0.074 |
| MRPS6 | +:chr21:34102834-34102908:34103496-34103519:34125340-34125480 | 0.226 | -0.007 | 0.051 |
| GOLGA4 | +:chr3:37251394-37251484:37266841-37266940:37273535-37273601 | 0.226 | 0.205 | -0.022 |
| TTC8 | +:chr14:88841036-88841196:88841424-88841557:88852970-88853056 | 0.225 | -0.074 | 0.029 |
| ZNF613 | +:chr19:51927219-51927540:51929731-51929896:51936027-51936235 | 0.224 | 0.1 | 0.038 |
| COL6A3 | -:chr2:237387581-237388184:237394586-237395204:237396726-237396847 | 0.223 | 0.228 | 0.056 |
| CAD | +:chr2:27240264-27240361:27240566-27240617:27240910-27240955 | 0.222 | 0.247 | 0.218 |
| NF2 | +:chr22:29681438-29681601:29683019-29683079:29694751-29694811 | 0.221 | 0.227 | -0.024 |
| FBXL12 | -:chr19:9810266-9811717:9813230-9813274:9818727-9819079 | 0.221 | 0.165 | -0.049 |
| PPHLN1 | +:chr12:42374862-42375074:42384939-42384996:42387455-42387535 | 0.221 | 0.148 | -0.015 |
| NDUFAF7 | +:chr2:37241577-37241791:37242634-37242693:37243862-37243973 | 0.218 | 0.05 | 0.087 |
| SPTAN1 | +:chr9:128609121-128609284:128609650-128609665:128611713-128611845 | 0.217 | 0.221 | 0.097 |

## Slide 29
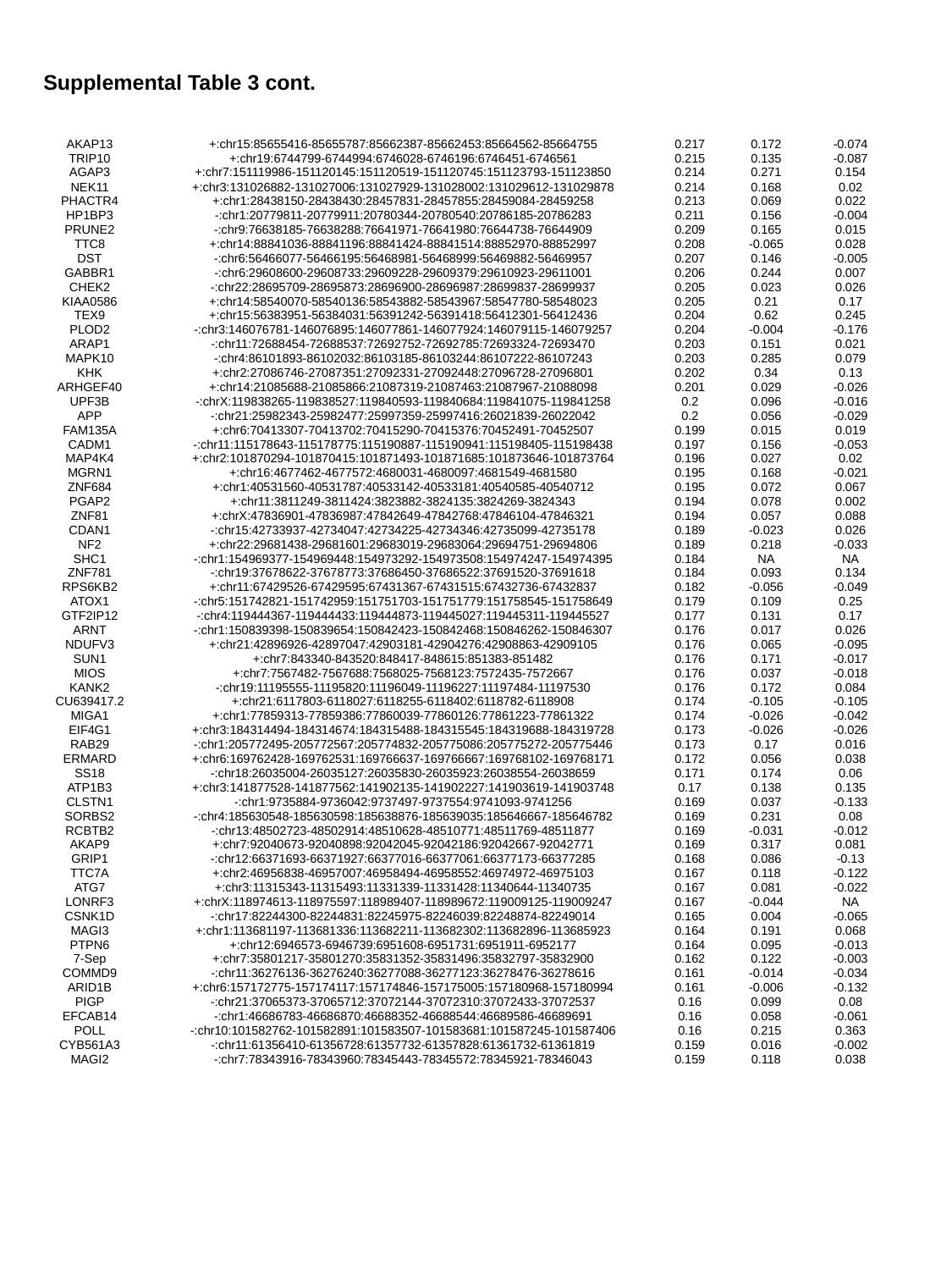

Supplemental Table 3 cont.
| AKAP13 | +:chr15:85655416-85655787:85662387-85662453:85664562-85664755 | 0.217 | 0.172 | -0.074 |
| --- | --- | --- | --- | --- |
| TRIP10 | +:chr19:6744799-6744994:6746028-6746196:6746451-6746561 | 0.215 | 0.135 | -0.087 |
| AGAP3 | +:chr7:151119986-151120145:151120519-151120745:151123793-151123850 | 0.214 | 0.271 | 0.154 |
| NEK11 | +:chr3:131026882-131027006:131027929-131028002:131029612-131029878 | 0.214 | 0.168 | 0.02 |
| PHACTR4 | +:chr1:28438150-28438430:28457831-28457855:28459084-28459258 | 0.213 | 0.069 | 0.022 |
| HP1BP3 | -:chr1:20779811-20779911:20780344-20780540:20786185-20786283 | 0.211 | 0.156 | -0.004 |
| PRUNE2 | -:chr9:76638185-76638288:76641971-76641980:76644738-76644909 | 0.209 | 0.165 | 0.015 |
| TTC8 | +:chr14:88841036-88841196:88841424-88841514:88852970-88852997 | 0.208 | -0.065 | 0.028 |
| DST | -:chr6:56466077-56466195:56468981-56468999:56469882-56469957 | 0.207 | 0.146 | -0.005 |
| GABBR1 | -:chr6:29608600-29608733:29609228-29609379:29610923-29611001 | 0.206 | 0.244 | 0.007 |
| CHEK2 | -:chr22:28695709-28695873:28696900-28696987:28699837-28699937 | 0.205 | 0.023 | 0.026 |
| KIAA0586 | +:chr14:58540070-58540136:58543882-58543967:58547780-58548023 | 0.205 | 0.21 | 0.17 |
| TEX9 | +:chr15:56383951-56384031:56391242-56391418:56412301-56412436 | 0.204 | 0.62 | 0.245 |
| PLOD2 | -:chr3:146076781-146076895:146077861-146077924:146079115-146079257 | 0.204 | -0.004 | -0.176 |
| ARAP1 | -:chr11:72688454-72688537:72692752-72692785:72693324-72693470 | 0.203 | 0.151 | 0.021 |
| MAPK10 | -:chr4:86101893-86102032:86103185-86103244:86107222-86107243 | 0.203 | 0.285 | 0.079 |
| KHK | +:chr2:27086746-27087351:27092331-27092448:27096728-27096801 | 0.202 | 0.34 | 0.13 |
| ARHGEF40 | +:chr14:21085688-21085866:21087319-21087463:21087967-21088098 | 0.201 | 0.029 | -0.026 |
| UPF3B | -:chrX:119838265-119838527:119840593-119840684:119841075-119841258 | 0.2 | 0.096 | -0.016 |
| APP | -:chr21:25982343-25982477:25997359-25997416:26021839-26022042 | 0.2 | 0.056 | -0.029 |
| FAM135A | +:chr6:70413307-70413702:70415290-70415376:70452491-70452507 | 0.199 | 0.015 | 0.019 |
| CADM1 | -:chr11:115178643-115178775:115190887-115190941:115198405-115198438 | 0.197 | 0.156 | -0.053 |
| MAP4K4 | +:chr2:101870294-101870415:101871493-101871685:101873646-101873764 | 0.196 | 0.027 | 0.02 |
| MGRN1 | +:chr16:4677462-4677572:4680031-4680097:4681549-4681580 | 0.195 | 0.168 | -0.021 |
| ZNF684 | +:chr1:40531560-40531787:40533142-40533181:40540585-40540712 | 0.195 | 0.072 | 0.067 |
| PGAP2 | +:chr11:3811249-3811424:3823882-3824135:3824269-3824343 | 0.194 | 0.078 | 0.002 |
| ZNF81 | +:chrX:47836901-47836987:47842649-47842768:47846104-47846321 | 0.194 | 0.057 | 0.088 |
| CDAN1 | -:chr15:42733937-42734047:42734225-42734346:42735099-42735178 | 0.189 | -0.023 | 0.026 |
| NF2 | +:chr22:29681438-29681601:29683019-29683064:29694751-29694806 | 0.189 | 0.218 | -0.033 |
| SHC1 | -:chr1:154969377-154969448:154973292-154973508:154974247-154974395 | 0.184 | NA | NA |
| ZNF781 | -:chr19:37678622-37678773:37686450-37686522:37691520-37691618 | 0.184 | 0.093 | 0.134 |
| RPS6KB2 | +:chr11:67429526-67429595:67431367-67431515:67432736-67432837 | 0.182 | -0.056 | -0.049 |
| ATOX1 | -:chr5:151742821-151742959:151751703-151751779:151758545-151758649 | 0.179 | 0.109 | 0.25 |
| GTF2IP12 | -:chr4:119444367-119444433:119444873-119445027:119445311-119445527 | 0.177 | 0.131 | 0.17 |
| ARNT | -:chr1:150839398-150839654:150842423-150842468:150846262-150846307 | 0.176 | 0.017 | 0.026 |
| NDUFV3 | +:chr21:42896926-42897047:42903181-42904276:42908863-42909105 | 0.176 | 0.065 | -0.095 |
| SUN1 | +:chr7:843340-843520:848417-848615:851383-851482 | 0.176 | 0.171 | -0.017 |
| MIOS | +:chr7:7567482-7567688:7568025-7568123:7572435-7572667 | 0.176 | 0.037 | -0.018 |
| KANK2 | -:chr19:11195555-11195820:11196049-11196227:11197484-11197530 | 0.176 | 0.172 | 0.084 |
| CU639417.2 | +:chr21:6117803-6118027:6118255-6118402:6118782-6118908 | 0.174 | -0.105 | -0.105 |
| MIGA1 | +:chr1:77859313-77859386:77860039-77860126:77861223-77861322 | 0.174 | -0.026 | -0.042 |
| EIF4G1 | +:chr3:184314494-184314674:184315488-184315545:184319688-184319728 | 0.173 | -0.026 | -0.026 |
| RAB29 | -:chr1:205772495-205772567:205774832-205775086:205775272-205775446 | 0.173 | 0.17 | 0.016 |
| ERMARD | +:chr6:169762428-169762531:169766637-169766667:169768102-169768171 | 0.172 | 0.056 | 0.038 |
| SS18 | -:chr18:26035004-26035127:26035830-26035923:26038554-26038659 | 0.171 | 0.174 | 0.06 |
| ATP1B3 | +:chr3:141877528-141877562:141902135-141902227:141903619-141903748 | 0.17 | 0.138 | 0.135 |
| CLSTN1 | -:chr1:9735884-9736042:9737497-9737554:9741093-9741256 | 0.169 | 0.037 | -0.133 |
| SORBS2 | -:chr4:185630548-185630598:185638876-185639035:185646667-185646782 | 0.169 | 0.231 | 0.08 |
| RCBTB2 | -:chr13:48502723-48502914:48510628-48510771:48511769-48511877 | 0.169 | -0.031 | -0.012 |
| AKAP9 | +:chr7:92040673-92040898:92042045-92042186:92042667-92042771 | 0.169 | 0.317 | 0.081 |
| GRIP1 | -:chr12:66371693-66371927:66377016-66377061:66377173-66377285 | 0.168 | 0.086 | -0.13 |
| TTC7A | +:chr2:46956838-46957007:46958494-46958552:46974972-46975103 | 0.167 | 0.118 | -0.122 |
| ATG7 | +:chr3:11315343-11315493:11331339-11331428:11340644-11340735 | 0.167 | 0.081 | -0.022 |
| LONRF3 | +:chrX:118974613-118975597:118989407-118989672:119009125-119009247 | 0.167 | -0.044 | NA |
| CSNK1D | -:chr17:82244300-82244831:82245975-82246039:82248874-82249014 | 0.165 | 0.004 | -0.065 |
| MAGI3 | +:chr1:113681197-113681336:113682211-113682302:113682896-113685923 | 0.164 | 0.191 | 0.068 |
| PTPN6 | +:chr12:6946573-6946739:6951608-6951731:6951911-6952177 | 0.164 | 0.095 | -0.013 |
| 7-Sep | +:chr7:35801217-35801270:35831352-35831496:35832797-35832900 | 0.162 | 0.122 | -0.003 |
| COMMD9 | -:chr11:36276136-36276240:36277088-36277123:36278476-36278616 | 0.161 | -0.014 | -0.034 |
| ARID1B | +:chr6:157172775-157174117:157174846-157175005:157180968-157180994 | 0.161 | -0.006 | -0.132 |
| PIGP | -:chr21:37065373-37065712:37072144-37072310:37072433-37072537 | 0.16 | 0.099 | 0.08 |
| EFCAB14 | -:chr1:46686783-46686870:46688352-46688544:46689586-46689691 | 0.16 | 0.058 | -0.061 |
| POLL | -:chr10:101582762-101582891:101583507-101583681:101587245-101587406 | 0.16 | 0.215 | 0.363 |
| CYB561A3 | -:chr11:61356410-61356728:61357732-61357828:61361732-61361819 | 0.159 | 0.016 | -0.002 |
| MAGI2 | -:chr7:78343916-78343960:78345443-78345572:78345921-78346043 | 0.159 | 0.118 | 0.038 |

## Slide 30
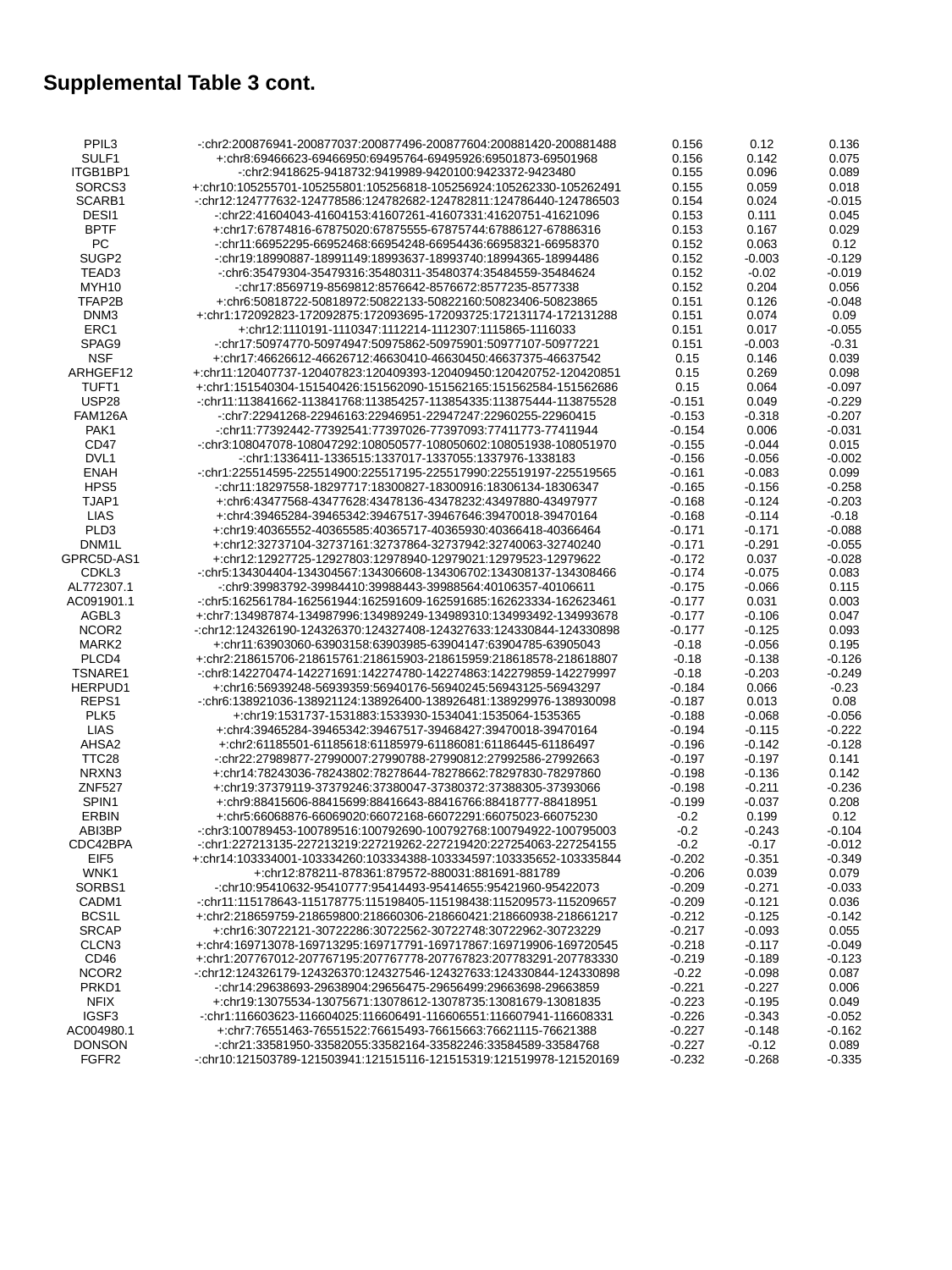

Supplemental Table 3 cont.
| PPIL3 | -:chr2:200876941-200877037:200877496-200877604:200881420-200881488 | 0.156 | 0.12 | 0.136 |
| --- | --- | --- | --- | --- |
| SULF1 | +:chr8:69466623-69466950:69495764-69495926:69501873-69501968 | 0.156 | 0.142 | 0.075 |
| ITGB1BP1 | -:chr2:9418625-9418732:9419989-9420100:9423372-9423480 | 0.155 | 0.096 | 0.089 |
| SORCS3 | +:chr10:105255701-105255801:105256818-105256924:105262330-105262491 | 0.155 | 0.059 | 0.018 |
| SCARB1 | -:chr12:124777632-124778586:124782682-124782811:124786440-124786503 | 0.154 | 0.024 | -0.015 |
| DESI1 | -:chr22:41604043-41604153:41607261-41607331:41620751-41621096 | 0.153 | 0.111 | 0.045 |
| BPTF | +:chr17:67874816-67875020:67875555-67875744:67886127-67886316 | 0.153 | 0.167 | 0.029 |
| PC | -:chr11:66952295-66952468:66954248-66954436:66958321-66958370 | 0.152 | 0.063 | 0.12 |
| SUGP2 | -:chr19:18990887-18991149:18993637-18993740:18994365-18994486 | 0.152 | -0.003 | -0.129 |
| TEAD3 | -:chr6:35479304-35479316:35480311-35480374:35484559-35484624 | 0.152 | -0.02 | -0.019 |
| MYH10 | -:chr17:8569719-8569812:8576642-8576672:8577235-8577338 | 0.152 | 0.204 | 0.056 |
| TFAP2B | +:chr6:50818722-50818972:50822133-50822160:50823406-50823865 | 0.151 | 0.126 | -0.048 |
| DNM3 | +:chr1:172092823-172092875:172093695-172093725:172131174-172131288 | 0.151 | 0.074 | 0.09 |
| ERC1 | +:chr12:1110191-1110347:1112214-1112307:1115865-1116033 | 0.151 | 0.017 | -0.055 |
| SPAG9 | -:chr17:50974770-50974947:50975862-50975901:50977107-50977221 | 0.151 | -0.003 | -0.31 |
| NSF | +:chr17:46626612-46626712:46630410-46630450:46637375-46637542 | 0.15 | 0.146 | 0.039 |
| ARHGEF12 | +:chr11:120407737-120407823:120409393-120409450:120420752-120420851 | 0.15 | 0.269 | 0.098 |
| TUFT1 | +:chr1:151540304-151540426:151562090-151562165:151562584-151562686 | 0.15 | 0.064 | -0.097 |
| USP28 | -:chr11:113841662-113841768:113854257-113854335:113875444-113875528 | -0.151 | 0.049 | -0.229 |
| FAM126A | -:chr7:22941268-22946163:22946951-22947247:22960255-22960415 | -0.153 | -0.318 | -0.207 |
| PAK1 | -:chr11:77392442-77392541:77397026-77397093:77411773-77411944 | -0.154 | 0.006 | -0.031 |
| CD47 | -:chr3:108047078-108047292:108050577-108050602:108051938-108051970 | -0.155 | -0.044 | 0.015 |
| DVL1 | -:chr1:1336411-1336515:1337017-1337055:1337976-1338183 | -0.156 | -0.056 | -0.002 |
| ENAH | -:chr1:225514595-225514900:225517195-225517990:225519197-225519565 | -0.161 | -0.083 | 0.099 |
| HPS5 | -:chr11:18297558-18297717:18300827-18300916:18306134-18306347 | -0.165 | -0.156 | -0.258 |
| TJAP1 | +:chr6:43477568-43477628:43478136-43478232:43497880-43497977 | -0.168 | -0.124 | -0.203 |
| LIAS | +:chr4:39465284-39465342:39467517-39467646:39470018-39470164 | -0.168 | -0.114 | -0.18 |
| PLD3 | +:chr19:40365552-40365585:40365717-40365930:40366418-40366464 | -0.171 | -0.171 | -0.088 |
| DNM1L | +:chr12:32737104-32737161:32737864-32737942:32740063-32740240 | -0.171 | -0.291 | -0.055 |
| GPRC5D-AS1 | +:chr12:12927725-12927803:12978940-12979021:12979523-12979622 | -0.172 | 0.037 | -0.028 |
| CDKL3 | -:chr5:134304404-134304567:134306608-134306702:134308137-134308466 | -0.174 | -0.075 | 0.083 |
| AL772307.1 | -:chr9:39983792-39984410:39988443-39988564:40106357-40106611 | -0.175 | -0.066 | 0.115 |
| AC091901.1 | -:chr5:162561784-162561944:162591609-162591685:162623334-162623461 | -0.177 | 0.031 | 0.003 |
| AGBL3 | +:chr7:134987874-134987996:134989249-134989310:134993492-134993678 | -0.177 | -0.106 | 0.047 |
| NCOR2 | -:chr12:124326190-124326370:124327408-124327633:124330844-124330898 | -0.177 | -0.125 | 0.093 |
| MARK2 | +:chr11:63903060-63903158:63903985-63904147:63904785-63905043 | -0.18 | -0.056 | 0.195 |
| PLCD4 | +:chr2:218615706-218615761:218615903-218615959:218618578-218618807 | -0.18 | -0.138 | -0.126 |
| TSNARE1 | -:chr8:142270474-142271691:142274780-142274863:142279859-142279997 | -0.18 | -0.203 | -0.249 |
| HERPUD1 | +:chr16:56939248-56939359:56940176-56940245:56943125-56943297 | -0.184 | 0.066 | -0.23 |
| REPS1 | -:chr6:138921036-138921124:138926400-138926481:138929976-138930098 | -0.187 | 0.013 | 0.08 |
| PLK5 | +:chr19:1531737-1531883:1533930-1534041:1535064-1535365 | -0.188 | -0.068 | -0.056 |
| LIAS | +:chr4:39465284-39465342:39467517-39468427:39470018-39470164 | -0.194 | -0.115 | -0.222 |
| AHSA2 | +:chr2:61185501-61185618:61185979-61186081:61186445-61186497 | -0.196 | -0.142 | -0.128 |
| TTC28 | -:chr22:27989877-27990007:27990788-27990812:27992586-27992663 | -0.197 | -0.197 | 0.141 |
| NRXN3 | +:chr14:78243036-78243802:78278644-78278662:78297830-78297860 | -0.198 | -0.136 | 0.142 |
| ZNF527 | +:chr19:37379119-37379246:37380047-37380372:37388305-37393066 | -0.198 | -0.211 | -0.236 |
| SPIN1 | +:chr9:88415606-88415699:88416643-88416766:88418777-88418951 | -0.199 | -0.037 | 0.208 |
| ERBIN | +:chr5:66068876-66069020:66072168-66072291:66075023-66075230 | -0.2 | 0.199 | 0.12 |
| ABI3BP | -:chr3:100789453-100789516:100792690-100792768:100794922-100795003 | -0.2 | -0.243 | -0.104 |
| CDC42BPA | -:chr1:227213135-227213219:227219262-227219420:227254063-227254155 | -0.2 | -0.17 | -0.012 |
| EIF5 | +:chr14:103334001-103334260:103334388-103334597:103335652-103335844 | -0.202 | -0.351 | -0.349 |
| WNK1 | +:chr12:878211-878361:879572-880031:881691-881789 | -0.206 | 0.039 | 0.079 |
| SORBS1 | -:chr10:95410632-95410777:95414493-95414655:95421960-95422073 | -0.209 | -0.271 | -0.033 |
| CADM1 | -:chr11:115178643-115178775:115198405-115198438:115209573-115209657 | -0.209 | -0.121 | 0.036 |
| BCS1L | +:chr2:218659759-218659800:218660306-218660421:218660938-218661217 | -0.212 | -0.125 | -0.142 |
| SRCAP | +:chr16:30722121-30722286:30722562-30722748:30722962-30723229 | -0.217 | -0.093 | 0.055 |
| CLCN3 | +:chr4:169713078-169713295:169717791-169717867:169719906-169720545 | -0.218 | -0.117 | -0.049 |
| CD46 | +:chr1:207767012-207767195:207767778-207767823:207783291-207783330 | -0.219 | -0.189 | -0.123 |
| NCOR2 | -:chr12:124326179-124326370:124327546-124327633:124330844-124330898 | -0.22 | -0.098 | 0.087 |
| PRKD1 | -:chr14:29638693-29638904:29656475-29656499:29663698-29663859 | -0.221 | -0.227 | 0.006 |
| NFIX | +:chr19:13075534-13075671:13078612-13078735:13081679-13081835 | -0.223 | -0.195 | 0.049 |
| IGSF3 | -:chr1:116603623-116604025:116606491-116606551:116607941-116608331 | -0.226 | -0.343 | -0.052 |
| AC004980.1 | +:chr7:76551463-76551522:76615493-76615663:76621115-76621388 | -0.227 | -0.148 | -0.162 |
| DONSON | -:chr21:33581950-33582055:33582164-33582246:33584589-33584768 | -0.227 | -0.12 | 0.089 |
| FGFR2 | -:chr10:121503789-121503941:121515116-121515319:121519978-121520169 | -0.232 | -0.268 | -0.335 |

## Slide 31
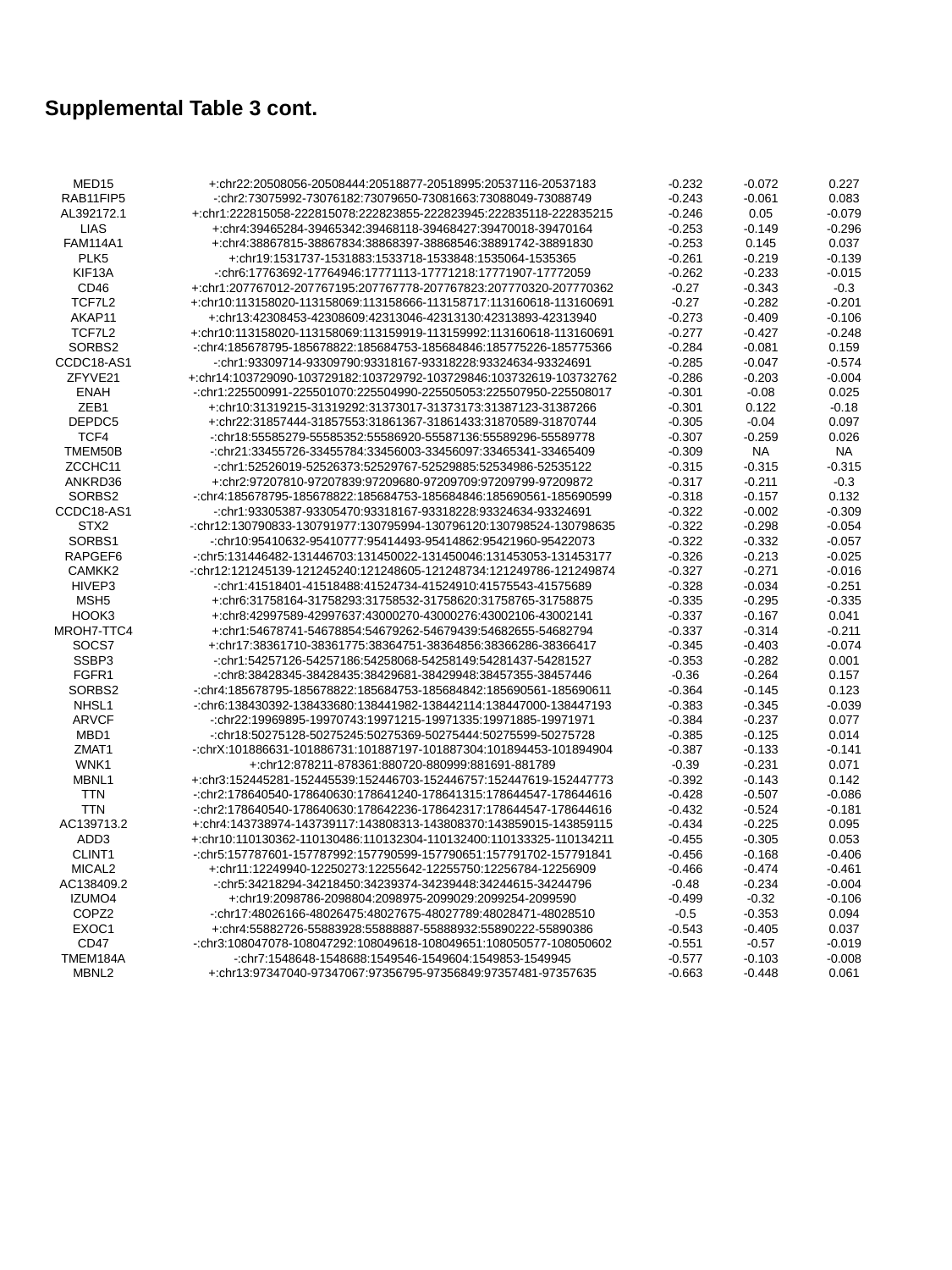

Supplemental Table 3 cont.
| MED15 | +:chr22:20508056-20508444:20518877-20518995:20537116-20537183 | -0.232 | -0.072 | 0.227 |
| --- | --- | --- | --- | --- |
| RAB11FIP5 | -:chr2:73075992-73076182:73079650-73081663:73088049-73088749 | -0.243 | -0.061 | 0.083 |
| AL392172.1 | +:chr1:222815058-222815078:222823855-222823945:222835118-222835215 | -0.246 | 0.05 | -0.079 |
| LIAS | +:chr4:39465284-39465342:39468118-39468427:39470018-39470164 | -0.253 | -0.149 | -0.296 |
| FAM114A1 | +:chr4:38867815-38867834:38868397-38868546:38891742-38891830 | -0.253 | 0.145 | 0.037 |
| PLK5 | +:chr19:1531737-1531883:1533718-1533848:1535064-1535365 | -0.261 | -0.219 | -0.139 |
| KIF13A | -:chr6:17763692-17764946:17771113-17771218:17771907-17772059 | -0.262 | -0.233 | -0.015 |
| CD46 | +:chr1:207767012-207767195:207767778-207767823:207770320-207770362 | -0.27 | -0.343 | -0.3 |
| TCF7L2 | +:chr10:113158020-113158069:113158666-113158717:113160618-113160691 | -0.27 | -0.282 | -0.201 |
| AKAP11 | +:chr13:42308453-42308609:42313046-42313130:42313893-42313940 | -0.273 | -0.409 | -0.106 |
| TCF7L2 | +:chr10:113158020-113158069:113159919-113159992:113160618-113160691 | -0.277 | -0.427 | -0.248 |
| SORBS2 | -:chr4:185678795-185678822:185684753-185684846:185775226-185775366 | -0.284 | -0.081 | 0.159 |
| CCDC18-AS1 | -:chr1:93309714-93309790:93318167-93318228:93324634-93324691 | -0.285 | -0.047 | -0.574 |
| ZFYVE21 | +:chr14:103729090-103729182:103729792-103729846:103732619-103732762 | -0.286 | -0.203 | -0.004 |
| ENAH | -:chr1:225500991-225501070:225504990-225505053:225507950-225508017 | -0.301 | -0.08 | 0.025 |
| ZEB1 | +:chr10:31319215-31319292:31373017-31373173:31387123-31387266 | -0.301 | 0.122 | -0.18 |
| DEPDC5 | +:chr22:31857444-31857553:31861367-31861433:31870589-31870744 | -0.305 | -0.04 | 0.097 |
| TCF4 | -:chr18:55585279-55585352:55586920-55587136:55589296-55589778 | -0.307 | -0.259 | 0.026 |
| TMEM50B | -:chr21:33455726-33455784:33456003-33456097:33465341-33465409 | -0.309 | NA | NA |
| ZCCHC11 | -:chr1:52526019-52526373:52529767-52529885:52534986-52535122 | -0.315 | -0.315 | -0.315 |
| ANKRD36 | +:chr2:97207810-97207839:97209680-97209709:97209799-97209872 | -0.317 | -0.211 | -0.3 |
| SORBS2 | -:chr4:185678795-185678822:185684753-185684846:185690561-185690599 | -0.318 | -0.157 | 0.132 |
| CCDC18-AS1 | -:chr1:93305387-93305470:93318167-93318228:93324634-93324691 | -0.322 | -0.002 | -0.309 |
| STX2 | -:chr12:130790833-130791977:130795994-130796120:130798524-130798635 | -0.322 | -0.298 | -0.054 |
| SORBS1 | -:chr10:95410632-95410777:95414493-95414862:95421960-95422073 | -0.322 | -0.332 | -0.057 |
| RAPGEF6 | -:chr5:131446482-131446703:131450022-131450046:131453053-131453177 | -0.326 | -0.213 | -0.025 |
| CAMKK2 | -:chr12:121245139-121245240:121248605-121248734:121249786-121249874 | -0.327 | -0.271 | -0.016 |
| HIVEP3 | -:chr1:41518401-41518488:41524734-41524910:41575543-41575689 | -0.328 | -0.034 | -0.251 |
| MSH5 | +:chr6:31758164-31758293:31758532-31758620:31758765-31758875 | -0.335 | -0.295 | -0.335 |
| HOOK3 | +:chr8:42997589-42997637:43000270-43000276:43002106-43002141 | -0.337 | -0.167 | 0.041 |
| MROH7-TTC4 | +:chr1:54678741-54678854:54679262-54679439:54682655-54682794 | -0.337 | -0.314 | -0.211 |
| SOCS7 | +:chr17:38361710-38361775:38364751-38364856:38366286-38366417 | -0.345 | -0.403 | -0.074 |
| SSBP3 | -:chr1:54257126-54257186:54258068-54258149:54281437-54281527 | -0.353 | -0.282 | 0.001 |
| FGFR1 | -:chr8:38428345-38428435:38429681-38429948:38457355-38457446 | -0.36 | -0.264 | 0.157 |
| SORBS2 | -:chr4:185678795-185678822:185684753-185684842:185690561-185690611 | -0.364 | -0.145 | 0.123 |
| NHSL1 | -:chr6:138430392-138433680:138441982-138442114:138447000-138447193 | -0.383 | -0.345 | -0.039 |
| ARVCF | -:chr22:19969895-19970743:19971215-19971335:19971885-19971971 | -0.384 | -0.237 | 0.077 |
| MBD1 | -:chr18:50275128-50275245:50275369-50275444:50275599-50275728 | -0.385 | -0.125 | 0.014 |
| ZMAT1 | -:chrX:101886631-101886731:101887197-101887304:101894453-101894904 | -0.387 | -0.133 | -0.141 |
| WNK1 | +:chr12:878211-878361:880720-880999:881691-881789 | -0.39 | -0.231 | 0.071 |
| MBNL1 | +:chr3:152445281-152445539:152446703-152446757:152447619-152447773 | -0.392 | -0.143 | 0.142 |
| TTN | -:chr2:178640540-178640630:178641240-178641315:178644547-178644616 | -0.428 | -0.507 | -0.086 |
| TTN | -:chr2:178640540-178640630:178642236-178642317:178644547-178644616 | -0.432 | -0.524 | -0.181 |
| AC139713.2 | +:chr4:143738974-143739117:143808313-143808370:143859015-143859115 | -0.434 | -0.225 | 0.095 |
| ADD3 | +:chr10:110130362-110130486:110132304-110132400:110133325-110134211 | -0.455 | -0.305 | 0.053 |
| CLINT1 | -:chr5:157787601-157787992:157790599-157790651:157791702-157791841 | -0.456 | -0.168 | -0.406 |
| MICAL2 | +:chr11:12249940-12250273:12255642-12255750:12256784-12256909 | -0.466 | -0.474 | -0.461 |
| AC138409.2 | -:chr5:34218294-34218450:34239374-34239448:34244615-34244796 | -0.48 | -0.234 | -0.004 |
| IZUMO4 | +:chr19:2098786-2098804:2098975-2099029:2099254-2099590 | -0.499 | -0.32 | -0.106 |
| COPZ2 | -:chr17:48026166-48026475:48027675-48027789:48028471-48028510 | -0.5 | -0.353 | 0.094 |
| EXOC1 | +:chr4:55882726-55883928:55888887-55888932:55890222-55890386 | -0.543 | -0.405 | 0.037 |
| CD47 | -:chr3:108047078-108047292:108049618-108049651:108050577-108050602 | -0.551 | -0.57 | -0.019 |
| TMEM184A | -:chr7:1548648-1548688:1549546-1549604:1549853-1549945 | -0.577 | -0.103 | -0.008 |
| MBNL2 | +:chr13:97347040-97347067:97356795-97356849:97357481-97357635 | -0.663 | -0.448 | 0.061 |

## Slide 32
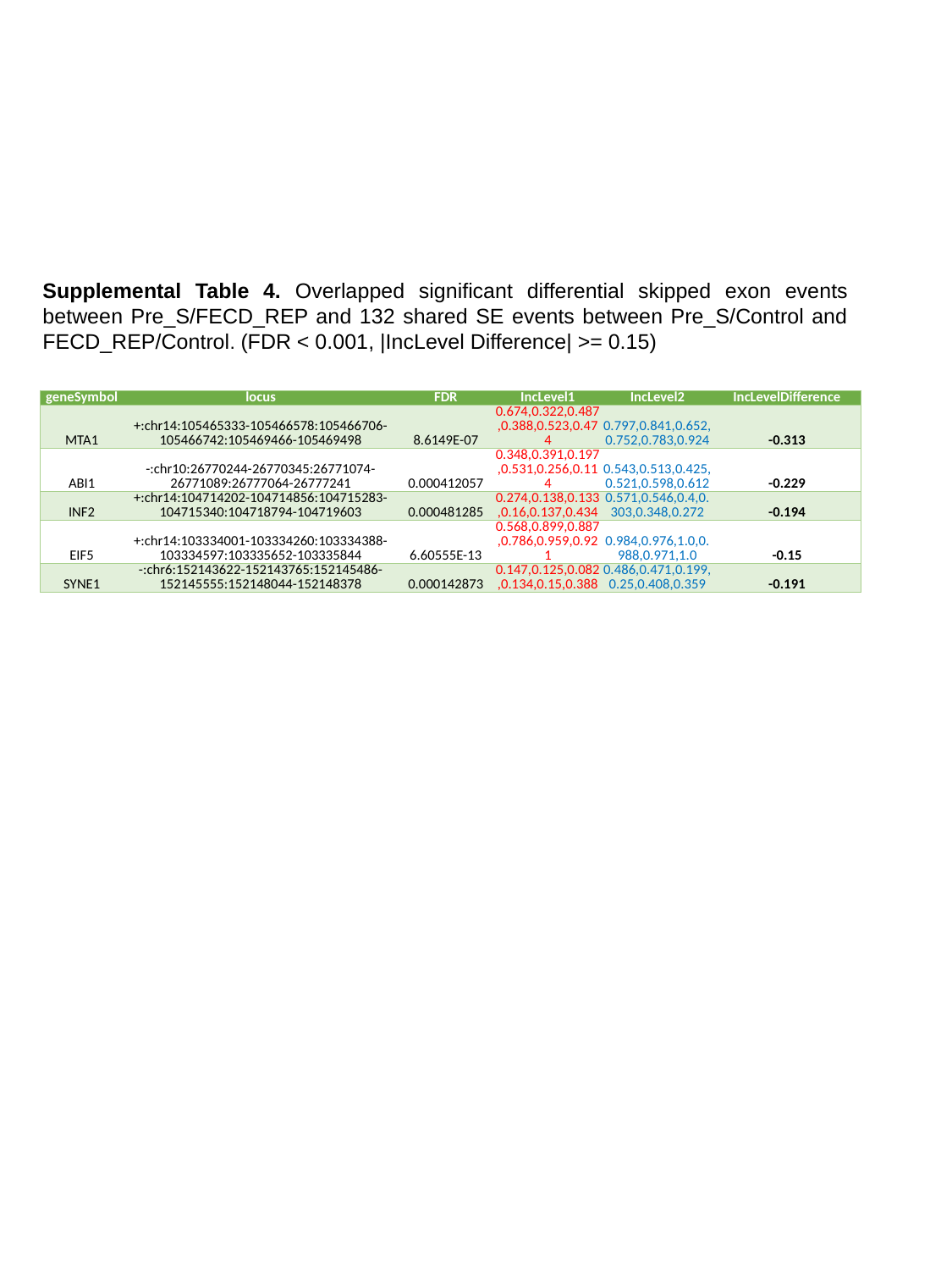

Supplemental Table 4. Overlapped significant differential skipped exon events between Pre_S/FECD_REP and 132 shared SE events between Pre_S/Control and FECD_REP/Control. (FDR < 0.001, |IncLevel Difference| >= 0.15)
| geneSymbol | locus | FDR | IncLevel1 | IncLevel2 | IncLevelDifference |
| --- | --- | --- | --- | --- | --- |
| MTA1 | +:chr14:105465333-105466578:105466706-105466742:105469466-105469498 | 8.6149E-07 | 0.674,0.322,0.487,0.388,0.523,0.474 | 0.797,0.841,0.652,0.752,0.783,0.924 | -0.313 |
| ABI1 | -:chr10:26770244-26770345:26771074-26771089:26777064-26777241 | 0.000412057 | 0.348,0.391,0.197,0.531,0.256,0.114 | 0.543,0.513,0.425,0.521,0.598,0.612 | -0.229 |
| INF2 | +:chr14:104714202-104714856:104715283-104715340:104718794-104719603 | 0.000481285 | 0.274,0.138,0.133,0.16,0.137,0.434 | 0.571,0.546,0.4,0.303,0.348,0.272 | -0.194 |
| EIF5 | +:chr14:103334001-103334260:103334388-103334597:103335652-103335844 | 6.60555E-13 | 0.568,0.899,0.887,0.786,0.959,0.921 | 0.984,0.976,1.0,0.988,0.971,1.0 | -0.15 |
| SYNE1 | -:chr6:152143622-152143765:152145486-152145555:152148044-152148378 | 0.000142873 | 0.147,0.125,0.082,0.134,0.15,0.388 | 0.486,0.471,0.199,0.25,0.408,0.359 | -0.191 |

## Slide 33
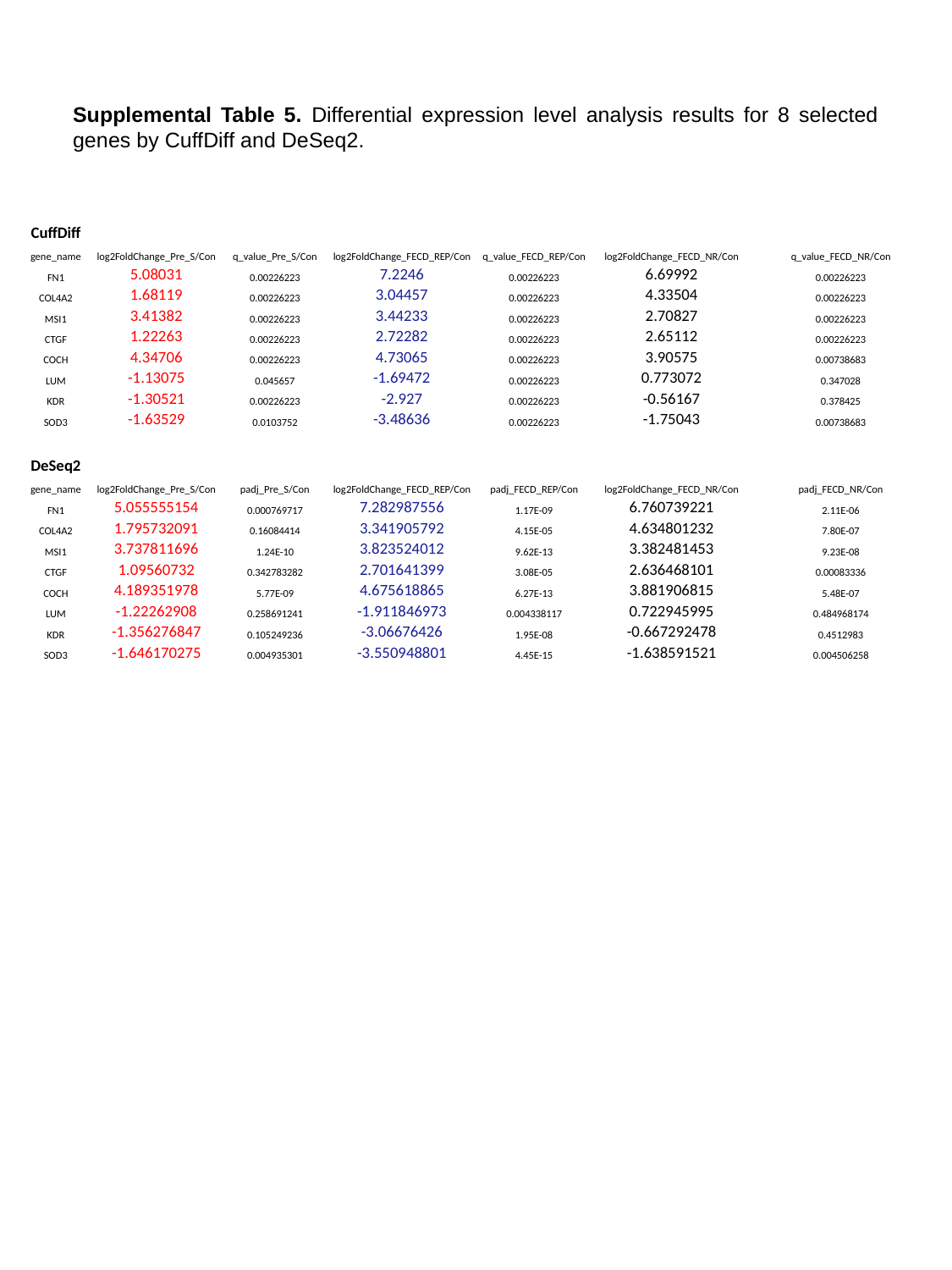

Supplemental Table 5. Differential expression level analysis results for 8 selected genes by CuffDiff and DeSeq2.
| CuffDiff | | | | | | |
| --- | --- | --- | --- | --- | --- | --- |
| gene\_name | log2FoldChange\_Pre\_S/Con | q\_value\_Pre\_S/Con | log2FoldChange\_FECD\_REP/Con | q\_value\_FECD\_REP/Con | log2FoldChange\_FECD\_NR/Con | q\_value\_FECD\_NR/Con |
| FN1 | 5.08031 | 0.00226223 | 7.2246 | 0.00226223 | 6.69992 | 0.00226223 |
| COL4A2 | 1.68119 | 0.00226223 | 3.04457 | 0.00226223 | 4.33504 | 0.00226223 |
| MSI1 | 3.41382 | 0.00226223 | 3.44233 | 0.00226223 | 2.70827 | 0.00226223 |
| CTGF | 1.22263 | 0.00226223 | 2.72282 | 0.00226223 | 2.65112 | 0.00226223 |
| COCH | 4.34706 | 0.00226223 | 4.73065 | 0.00226223 | 3.90575 | 0.00738683 |
| LUM | -1.13075 | 0.045657 | -1.69472 | 0.00226223 | 0.773072 | 0.347028 |
| KDR | -1.30521 | 0.00226223 | -2.927 | 0.00226223 | -0.56167 | 0.378425 |
| SOD3 | -1.63529 | 0.0103752 | -3.48636 | 0.00226223 | -1.75043 | 0.00738683 |
| | | | | | | |
| DeSeq2 | | | | | | |
| gene\_name | log2FoldChange\_Pre\_S/Con | padj\_Pre\_S/Con | log2FoldChange\_FECD\_REP/Con | padj\_FECD\_REP/Con | log2FoldChange\_FECD\_NR/Con | padj\_FECD\_NR/Con |
| FN1 | 5.055555154 | 0.000769717 | 7.282987556 | 1.17E-09 | 6.760739221 | 2.11E-06 |
| COL4A2 | 1.795732091 | 0.16084414 | 3.341905792 | 4.15E-05 | 4.634801232 | 7.80E-07 |
| MSI1 | 3.737811696 | 1.24E-10 | 3.823524012 | 9.62E-13 | 3.382481453 | 9.23E-08 |
| CTGF | 1.09560732 | 0.342783282 | 2.701641399 | 3.08E-05 | 2.636468101 | 0.00083336 |
| COCH | 4.189351978 | 5.77E-09 | 4.675618865 | 6.27E-13 | 3.881906815 | 5.48E-07 |
| LUM | -1.22262908 | 0.258691241 | -1.911846973 | 0.004338117 | 0.722945995 | 0.484968174 |
| KDR | -1.356276847 | 0.105249236 | -3.06676426 | 1.95E-08 | -0.667292478 | 0.4512983 |
| SOD3 | -1.646170275 | 0.004935301 | -3.550948801 | 4.45E-15 | -1.638591521 | 0.004506258 |

## Slide 34
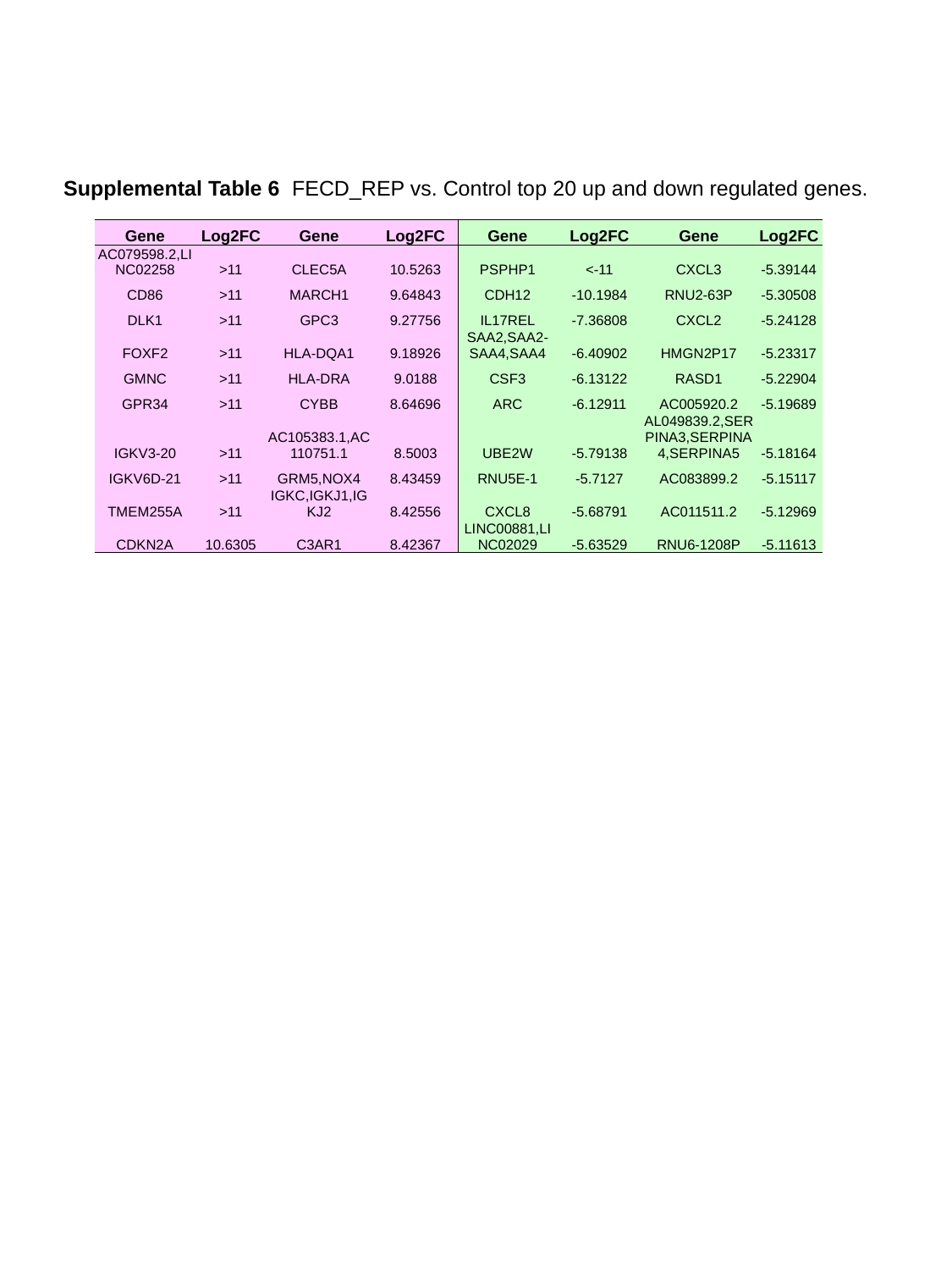

Supplemental Table 6 FECD_REP vs. Control top 20 up and down regulated genes.
| Gene | Log2FC | Gene | Log2FC | Gene | Log2FC | Gene | Log2FC |
| --- | --- | --- | --- | --- | --- | --- | --- |
| AC079598.2,LINC02258 | >11 | CLEC5A | 10.5263 | PSPHP1 | <-11 | CXCL3 | -5.39144 |
| CD86 | >11 | MARCH1 | 9.64843 | CDH12 | -10.1984 | RNU2-63P | -5.30508 |
| DLK1 | >11 | GPC3 | 9.27756 | IL17REL | -7.36808 | CXCL2 | -5.24128 |
| FOXF2 | >11 | HLA-DQA1 | 9.18926 | SAA2,SAA2-SAA4,SAA4 | -6.40902 | HMGN2P17 | -5.23317 |
| GMNC | >11 | HLA-DRA | 9.0188 | CSF3 | -6.13122 | RASD1 | -5.22904 |
| GPR34 | >11 | CYBB | 8.64696 | ARC | -6.12911 | AC005920.2 | -5.19689 |
| IGKV3-20 | >11 | AC105383.1,AC110751.1 | 8.5003 | UBE2W | -5.79138 | AL049839.2,SERPINA3,SERPINA4,SERPINA5 | -5.18164 |
| IGKV6D-21 | >11 | GRM5,NOX4 | 8.43459 | RNU5E-1 | -5.7127 | AC083899.2 | -5.15117 |
| TMEM255A | >11 | IGKC,IGKJ1,IGKJ2 | 8.42556 | CXCL8 | -5.68791 | AC011511.2 | -5.12969 |
| CDKN2A | 10.6305 | C3AR1 | 8.42367 | LINC00881,LINC02029 | -5.63529 | RNU6-1208P | -5.11613 |

## Slide 35
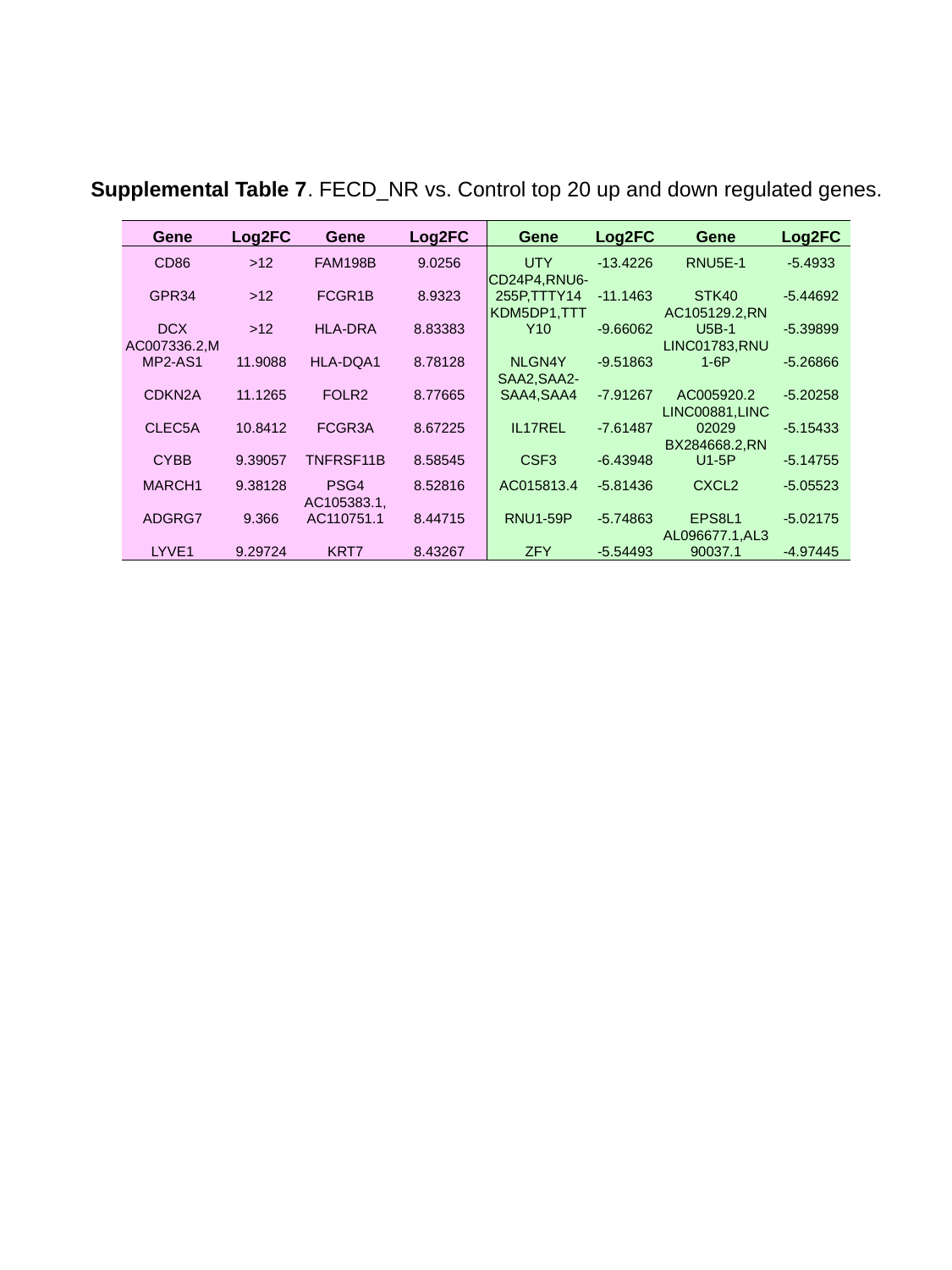

Supplemental Table 7. FECD_NR vs. Control top 20 up and down regulated genes.
| Gene | Log2FC | Gene | Log2FC | Gene | Log2FC | Gene | Log2FC |
| --- | --- | --- | --- | --- | --- | --- | --- |
| CD86 | >12 | FAM198B | 9.0256 | UTY | -13.4226 | RNU5E-1 | -5.4933 |
| GPR34 | >12 | FCGR1B | 8.9323 | CD24P4,RNU6-255P,TTTY14 | -11.1463 | STK40 | -5.44692 |
| DCX | >12 | HLA-DRA | 8.83383 | KDM5DP1,TTTY10 | -9.66062 | AC105129.2,RNU5B-1 | -5.39899 |
| AC007336.2,MMP2-AS1 | 11.9088 | HLA-DQA1 | 8.78128 | NLGN4Y | -9.51863 | LINC01783,RNU1-6P | -5.26866 |
| CDKN2A | 11.1265 | FOLR2 | 8.77665 | SAA2,SAA2-SAA4,SAA4 | -7.91267 | AC005920.2 | -5.20258 |
| CLEC5A | 10.8412 | FCGR3A | 8.67225 | IL17REL | -7.61487 | LINC00881,LINC02029 | -5.15433 |
| CYBB | 9.39057 | TNFRSF11B | 8.58545 | CSF3 | -6.43948 | BX284668.2,RNU1-5P | -5.14755 |
| MARCH1 | 9.38128 | PSG4 | 8.52816 | AC015813.4 | -5.81436 | CXCL2 | -5.05523 |
| ADGRG7 | 9.366 | AC105383.1,AC110751.1 | 8.44715 | RNU1-59P | -5.74863 | EPS8L1 | -5.02175 |
| LYVE1 | 9.29724 | KRT7 | 8.43267 | ZFY | -5.54493 | AL096677.1,AL390037.1 | -4.97445 |

## Slide 36
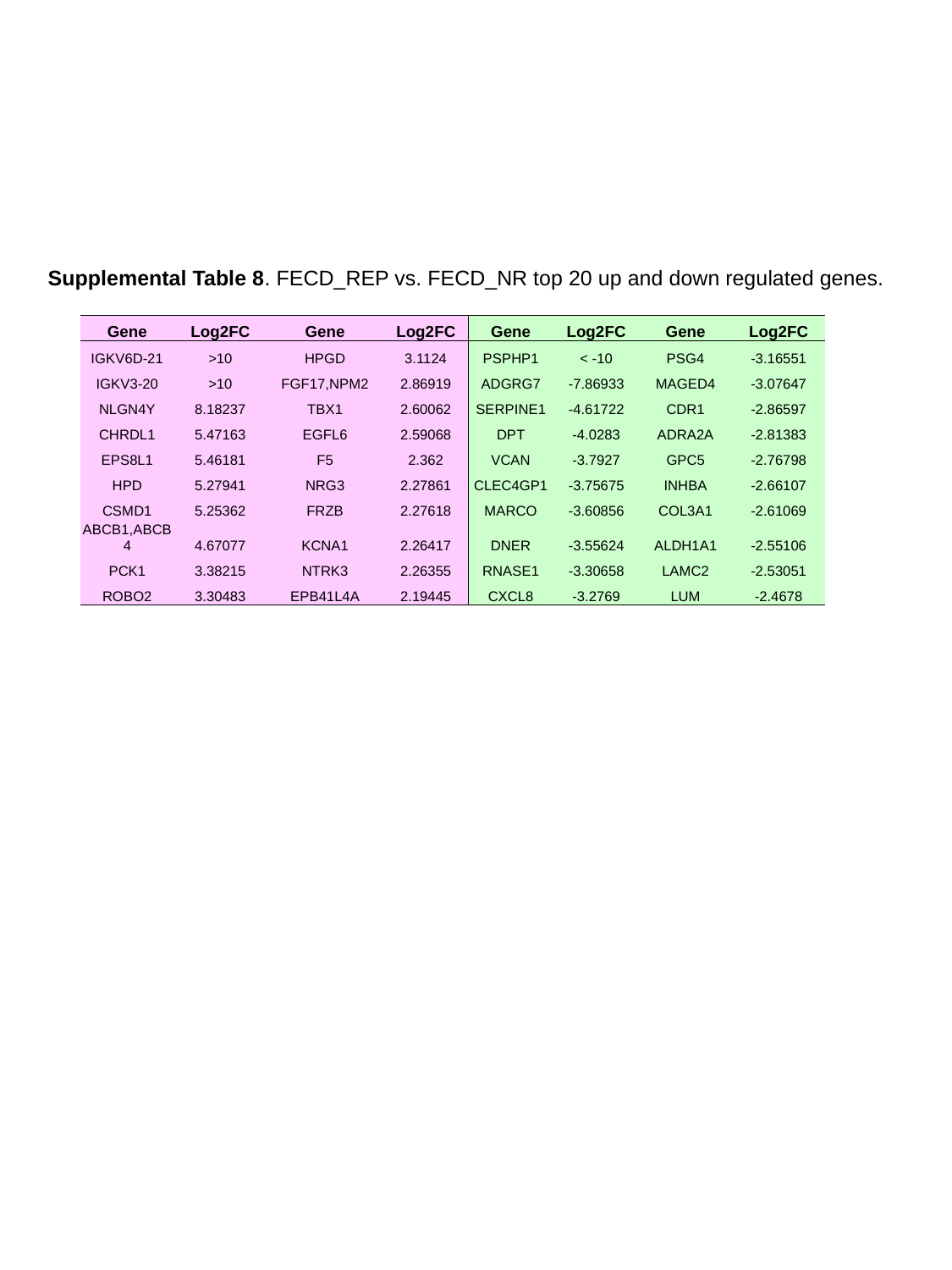

Supplemental Table 8. FECD_REP vs. FECD_NR top 20 up and down regulated genes.
| Gene | Log2FC | Gene | Log2FC | Gene | Log2FC | Gene | Log2FC |
| --- | --- | --- | --- | --- | --- | --- | --- |
| IGKV6D-21 | >10 | HPGD | 3.1124 | PSPHP1 | < -10 | PSG4 | -3.16551 |
| IGKV3-20 | >10 | FGF17,NPM2 | 2.86919 | ADGRG7 | -7.86933 | MAGED4 | -3.07647 |
| NLGN4Y | 8.18237 | TBX1 | 2.60062 | SERPINE1 | -4.61722 | CDR1 | -2.86597 |
| CHRDL1 | 5.47163 | EGFL6 | 2.59068 | DPT | -4.0283 | ADRA2A | -2.81383 |
| EPS8L1 | 5.46181 | F5 | 2.362 | VCAN | -3.7927 | GPC5 | -2.76798 |
| HPD | 5.27941 | NRG3 | 2.27861 | CLEC4GP1 | -3.75675 | INHBA | -2.66107 |
| CSMD1 | 5.25362 | FRZB | 2.27618 | MARCO | -3.60856 | COL3A1 | -2.61069 |
| ABCB1,ABCB4 | 4.67077 | KCNA1 | 2.26417 | DNER | -3.55624 | ALDH1A1 | -2.55106 |
| PCK1 | 3.38215 | NTRK3 | 2.26355 | RNASE1 | -3.30658 | LAMC2 | -2.53051 |
| ROBO2 | 3.30483 | EPB41L4A | 2.19445 | CXCL8 | -3.2769 | LUM | -2.4678 |

## Slide 37
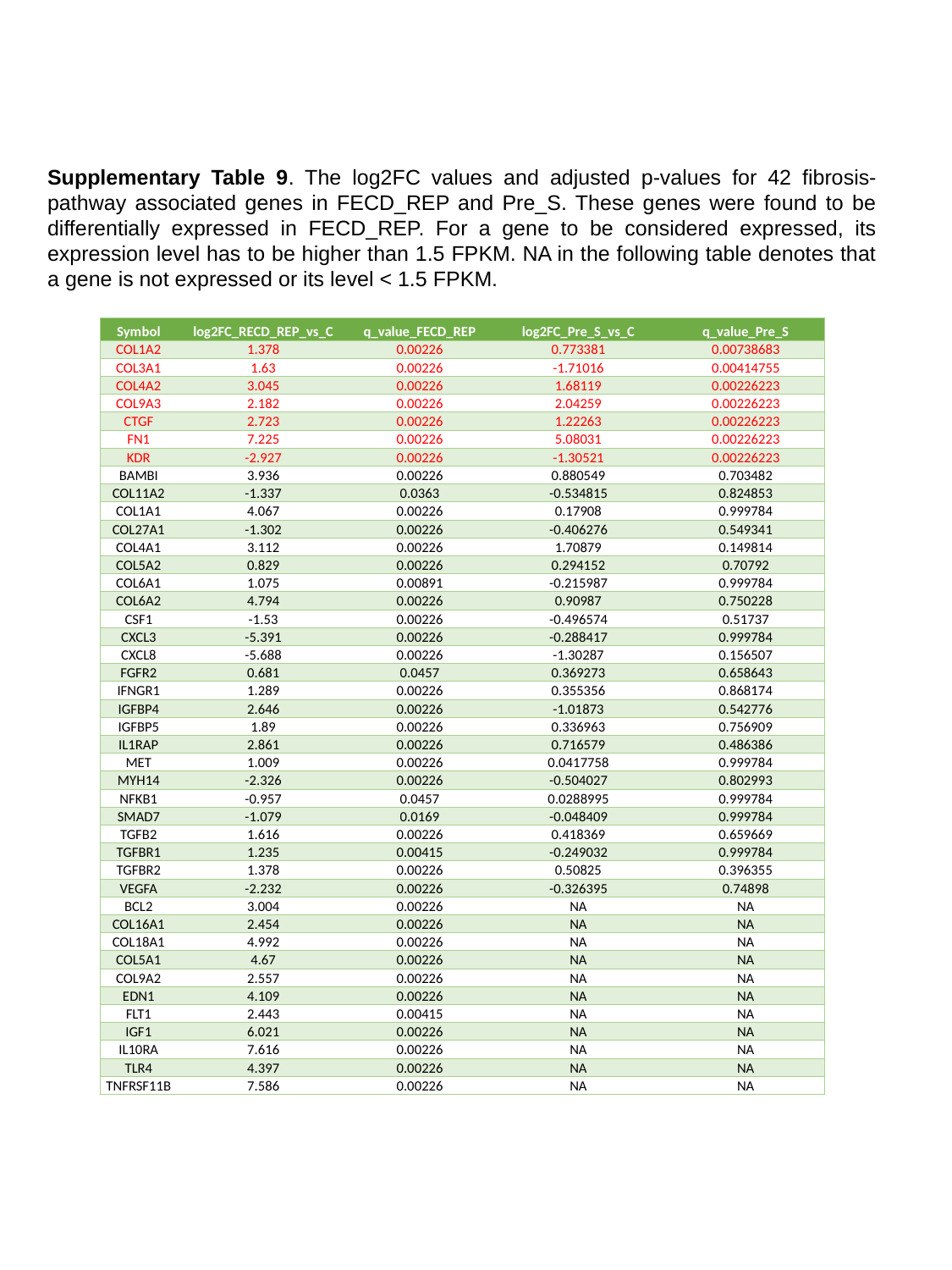

Supplementary Table 9. The log2FC values and adjusted p-values for 42 fibrosis-pathway associated genes in FECD_REP and Pre_S. These genes were found to be differentially expressed in FECD_REP. For a gene to be considered expressed, its expression level has to be higher than 1.5 FPKM. NA in the following table denotes that a gene is not expressed or its level < 1.5 FPKM.
| Symbol | log2FC\_RECD\_REP\_vs\_C | q\_value\_FECD\_REP | log2FC\_Pre\_S\_vs\_C | q\_value\_Pre\_S |
| --- | --- | --- | --- | --- |
| COL1A2 | 1.378 | 0.00226 | 0.773381 | 0.00738683 |
| COL3A1 | 1.63 | 0.00226 | -1.71016 | 0.00414755 |
| COL4A2 | 3.045 | 0.00226 | 1.68119 | 0.00226223 |
| COL9A3 | 2.182 | 0.00226 | 2.04259 | 0.00226223 |
| CTGF | 2.723 | 0.00226 | 1.22263 | 0.00226223 |
| FN1 | 7.225 | 0.00226 | 5.08031 | 0.00226223 |
| KDR | -2.927 | 0.00226 | -1.30521 | 0.00226223 |
| BAMBI | 3.936 | 0.00226 | 0.880549 | 0.703482 |
| COL11A2 | -1.337 | 0.0363 | -0.534815 | 0.824853 |
| COL1A1 | 4.067 | 0.00226 | 0.17908 | 0.999784 |
| COL27A1 | -1.302 | 0.00226 | -0.406276 | 0.549341 |
| COL4A1 | 3.112 | 0.00226 | 1.70879 | 0.149814 |
| COL5A2 | 0.829 | 0.00226 | 0.294152 | 0.70792 |
| COL6A1 | 1.075 | 0.00891 | -0.215987 | 0.999784 |
| COL6A2 | 4.794 | 0.00226 | 0.90987 | 0.750228 |
| CSF1 | -1.53 | 0.00226 | -0.496574 | 0.51737 |
| CXCL3 | -5.391 | 0.00226 | -0.288417 | 0.999784 |
| CXCL8 | -5.688 | 0.00226 | -1.30287 | 0.156507 |
| FGFR2 | 0.681 | 0.0457 | 0.369273 | 0.658643 |
| IFNGR1 | 1.289 | 0.00226 | 0.355356 | 0.868174 |
| IGFBP4 | 2.646 | 0.00226 | -1.01873 | 0.542776 |
| IGFBP5 | 1.89 | 0.00226 | 0.336963 | 0.756909 |
| IL1RAP | 2.861 | 0.00226 | 0.716579 | 0.486386 |
| MET | 1.009 | 0.00226 | 0.0417758 | 0.999784 |
| MYH14 | -2.326 | 0.00226 | -0.504027 | 0.802993 |
| NFKB1 | -0.957 | 0.0457 | 0.0288995 | 0.999784 |
| SMAD7 | -1.079 | 0.0169 | -0.048409 | 0.999784 |
| TGFB2 | 1.616 | 0.00226 | 0.418369 | 0.659669 |
| TGFBR1 | 1.235 | 0.00415 | -0.249032 | 0.999784 |
| TGFBR2 | 1.378 | 0.00226 | 0.50825 | 0.396355 |
| VEGFA | -2.232 | 0.00226 | -0.326395 | 0.74898 |
| BCL2 | 3.004 | 0.00226 | NA | NA |
| COL16A1 | 2.454 | 0.00226 | NA | NA |
| COL18A1 | 4.992 | 0.00226 | NA | NA |
| COL5A1 | 4.67 | 0.00226 | NA | NA |
| COL9A2 | 2.557 | 0.00226 | NA | NA |
| EDN1 | 4.109 | 0.00226 | NA | NA |
| FLT1 | 2.443 | 0.00415 | NA | NA |
| IGF1 | 6.021 | 0.00226 | NA | NA |
| IL10RA | 7.616 | 0.00226 | NA | NA |
| TLR4 | 4.397 | 0.00226 | NA | NA |
| TNFRSF11B | 7.586 | 0.00226 | NA | NA |
